# Human Purkinje in silico model enables mechanistic investigations into automaticity and pro-arrhythmic abnormalities

**DOI:** 10.1016/j.yjmcc.2020.04.001

**Published:** 2020-05

**Authors:** Cristian Trovato, Elisa Passini, Norbert Nagy, András Varró, Najah Abi-Gerges, Stefano Severi, Blanca Rodriguez

**Affiliations:** a Department of Computer Science, British Heart Foundation Centre of Research Excellence, University of Oxford, Oxford OX13QD, United Kingdom; b Department of Pharmacology and Pharmacotherapy, University of Szeged, Szeged H-6720, Hungary; c AnaBios Corporation, San Diego Science Center, San Diego, CA 92109, USA; d Department of Electrical, Electronic and Information Engineering, University of Bologna, Cesena 47521, Italy; e Department of Pharmacology and Pharmacotherapy, Interdisciplinary Excellence Centre, University of Szeged, Szeged, Hungary

**Keywords:** Cardiac Purkinje, Human, Electrophysiology, Arrhythmias, In silico trials, Computer modeling, AP(s), action potential(s), APA, action potential amplitude, APD_x_, AP duration at X% of repolarisation, BCL, basic cycle length, DAD(s), delayed after-depolarisation(s), DMP, diastolic membrane potential, dV/dt_MAX_, maximum upstroke velocity, EAD(s), early after-depolarisation(s), EOP, membrane potential at the end of repolarisation, G_X_, I_X_ conductance, IC_50_, concentration for 50% channel inhibition, I_CaL_, L-type Ca^2+^ current, I_CaT_, T-type Ca^2+^ current, I_f_, funny current, I_K1_, inward rectifier K^+^ current, I_Kr_, rapid delayed rectifier K^+^ current, I_Ks_, slow delayed rectifier K^+^ current, I_Na_, fast Na^+^ current, I_NaK_, Na^+^-K^+^ pump current, I_NaL_, late Na^+^ current, I_NCX_, Na^+^-Ca^2+^ exchanger current, I_to_, transient outward K^+^ current, I_sus_, sustained outward K^+^ current, ORd, O'Hara-Rudy dynamic human ventricular model, PC(s), Purkinje cells, PRd, Pan Li-Rudy dynamic canine Purkinje model, SS, steady state, TOP, take-off potential (membrane potential before depolarisation), Trovato2020, new human cardiac Purkinje AP model, V_m_, membrane potential

## Abstract

Cardiac Purkinje cells (PCs) are implicated in lethal arrhythmias caused by cardiac diseases, mutations, and drug action. However, the pro-arrhythmic mechanisms in PCs are not entirely understood, particularly in humans, as most investigations are conducted in animals. The aims of this study are to present a novel human PCs electrophysiology biophysically-detailed computational model, and to disentangle ionic mechanisms of human Purkinje-related electrophysiology, pacemaker activity and arrhythmogenicity. The new Trovato2020 model incorporates detailed Purkinje-specific ionic currents and Ca^2+^ handling, and was developed, calibrated and validated using human experimental data acquired at multiple frequencies, both in control conditions and following drug application. Multiscale investigations were performed in a Purkinje cell, in fibre and using an experimentally-calibrated population of PCs to evaluate biological variability. Simulations demonstrate the human Purkinje Trovato2020 model is the first one to yield: (i) all key AP features consistent with human Purkinje recordings; (ii) Automaticity with funny current up-regulation (iii) EADs at slow pacing and with 85% hERG block; (iv) DADs following fast pacing; (v) conduction velocity of 160 cm/s in a Purkinje fibre, as reported in human. The human in silico PCs population highlights that: (1) EADs are caused by I_CaL_ reactivation in PCs with large inward currents; (2) DADs and triggered APs occur in PCs experiencing Ca^2+^ accumulation, at fast pacing, caused by large L-type calcium current and small Na^+^/Ca^2+^ exchanger. The novel human Purkinje model unlocks further investigations into the role of cardiac Purkinje in ventricular arrhythmias through computer modeling and multiscale simulations.

## Highlights

• A human in silico AP model was developed to investigate arrhythmia in cardiac Purkinje.
• The new Purkinje model enables multiscale investigations from single cell to tissue.
• Populations of human Purkinje models reproduce and explain experimental variability.
• Ca^2+^-current reactivation triggers EADs in virtual Purkinje cells with weak repolarisation reserve.
• Ca^2+^ accumulation caused by increased Ca^2+^ and NCX currents triggers DADs.

## Graphical abstract

**Figure:**
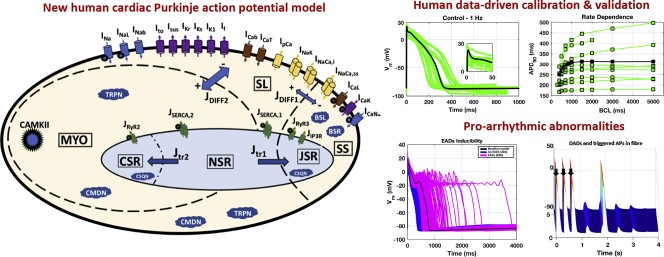

## 1 Introduction

Cardiac Purkinje cells (PCs) play a crucial role in ventricular excitation since they guarantee a correct excitation pattern and therefore a synchronised sequence of cardiac contraction. Mounting evidence identifies PCs as an important trigger of arrhythmias [1]. Particularly, PCs may be involved in the generation of Torsade de Pointes arrhythmias [2], associated with the long QT syndrome [3], either genetic or drug-induced. Indeed, PCs obtained often from dog or rabbit hearts are a cardiac preparation commonly used for preclinical cardiotoxicity screening [4].

Purkinje and ventricular cardiomyocytes are different both in structure and electrophysiology. The main structural difference is the low density of t-tubuli in PCs [5], which induces spatial heterogeneity of Ca^2+^ cycling [6]. The action potential (AP) of PCs is characterised by a faster depolarisation phase, a more negative plateau and a longer AP duration (APD) compared to ventricular APs [1,7]. Due to their longer APD, PCs may be more prone than ventricular myocytes to develop pro-arrhythmic abnormalities, i.e. early and delayed after-depolarisation (EADs and DADs, respectively) [2]. Interestingly, experimental studies have reported that PCs with a less negative diastolic membrane potential (DMP) show automaticity, whereas, well-polarised PCs do not [7,8].

Isolation of PCs requires challenging procedures which make the investigation of the electrophysiological properties of PCs difficult compared to other types of cardiomyocytes [9]. Furthermore, experiments on PCs are not usually conducted in human but rather in animal models such as guinea pig, rabbit, dog, cow, sheep, all exhibiting significant interspecies differences in structure, electrophysiology and calcium handling [[10], [11], [12]]. Thus, mechanistic complexity, limited access to human tissue and experimental and ethical constraints impair our understanding of the ionic mechanisms and contribution to human arrhythmias of the Purkinje system.

The goals of our study are to integrate current knowledge on human PC electrophysiology through the development of the novel computational Trovato2020 model, and to investigate mechanisms of pro-arrhythmic abnormalities in human PC through cellular and tissue simulation studies. Human Purkinje voltage-clamp data [9], novel and partly published AP measurements [13], as well as information from the literature were used to develop, calibrate and validate the Trovato2020 model. The model structure incorporates Purkinje-specific ionic currents and a detailed Ca^2+^ subsystem, as in a recently published canine model (PRd, [14]), which were not considered in previously published human Purkinje-based computational models (STW [15]; TT08 [16]; SMP [17]). Simulations with the new Trovato2020 model: i) reproduce experimental recordings in a wide range of protocols as well as electrophysiological alterations following selective hERG and Ca^2+^ channels blocks; ii) explain the ionic mechanisms underlying pro-arrhythmic abnormalities and automaticity. Biological variability was also studied through the construction and evaluation of a populations of models [18,19] to investigate and explain the mechanisms underlying EADs, DADs and triggered activity in human Purkinje cardiomyocytes. Electrical propagation was successfully simulated in a human Purkinje fibre. The Trovato2020 model is available on the CellML repository (www.cellml.org) as well as in several formats (Matlab, C++ and Fortran) in the Oxford Computational Cardiovascular Science Team website (www.cs.ox.ac.uk/ccs).

## 2 Materials and methods

### 2.1 Experimental data

Three different experimental datasets were used in this study for calibration, optimisation and validation of the novel human cardiac Purkinje electrophysiology Trovato2020 model:

• Dataset I. Ionic current recordings from [9]. These consist of the I-V curves for the transient and sustained outward potassium currents (I_to_ and I_sus_, respectively) and for the inward potassium rectifier current (I_K1_), as well as I_to_ steady state inactivation/activation curves, and inactivation time constants. Data were acquired from *n* = 20 PCs isolated from free-running false tendons from *N* = 9 failing human hearts.
• Dataset II. AP recordings from *n* = 17 Purkinje fibres from *N* = 7 undiseased human hearts, acquired at multiple frequencies - basic cycle length (BCL) from 400 to 5000 ms - partly published in [13]. AP recordings were analysed to extract the following 9 biomarkers: maximum upstroke velocity (dV/dt_MAX_); AP duration (APD) at 90%, 75%, 50%, 25% and 10% of repolarisation (APD_90,_ APD_75,_ APD_50,_ APD_25,_ APD_10_); AP Amplitude (APA); membrane potential before depolarisation (TOP, take-off potential); membrane potential at the end of repolarisation (EOP, end of potential). Table 1 shows the minimum and maximum experimental values for each biomarker and BCL. A graphical visualisation of the data is presented in Fig. S1 (Supplementary Material).

**Table 1:** Experimental AP biomarkers.

	BCL (ms)															
	400		500		700		1000		1500		2000		3000		5000	
	Min	Max	Min	Max	Min	Max	Min	Max	Min	Max	Min	Max	Min	Max	Min	Max
dV/dt_Max_ (V/s)	242	576	176	674	176	644	154	688	195	605	195	625	195	625	151	625
APD_90_ (ms)	167	288	173	338	175	389	187	438	177	445	178	415	179	470	180	497
APD_75_ (ms)	146	253	150	298	151	346	166	384	155	394	157	371	157	421	159	453
APD_50_ (ms)	114	199	118	246	121	292	124	349	122	339	127	292	125	365	129	392
APD_25_ (ms)	32	137	39	148	45	167	31	185	34	209	20	147	19	218	25	226
APD_10_ (ms)	2	65	1.4	63	1	82	1	107	1	75	1	23	1	86	1	91
APA (mV)	101	115	101	115	91	115	93	116	93	122	92	115	94	123	89	115
TOP (mV)	−88	−80	−89	−75	−90	−76	−91	−79	−92	−77	−87	−76	−93	−77	−88	−73
EOP (mV)	−91	−85	−89	−79	−90	−75	−94	−81	−93	−77	−89	−77	−93	−77	−88	−72

Minimum and maximum experimental values for the 9 AP biomarkers recorded in human cardiac Purkinje cells at multiple BCL: 400 ms (*n* = 7), 500 ms (*n* = 7), 700 ms (*n* = 9), 1000 (*n* = 17), 1500 ms (*n* = 9), 2000 ms (*n* = 7), 3000 (*n* = 8), 5000 ms (*n* = 8).

**BCL:** Basic cycle length; **dV/dt**_**MAX**_**:** maximum upstroke velocity; **APD**_**x**_**:** AP duration at X% of repolarisation; **APA:** action potential amplitude; **TOP**: membrane potential before depolarisation; **EOP:** membrane potential at the end of repolarisation.
• Dataset III. AP recordings from *n* = 3 Purkinje fibres from *N* = 3 undiseased human hearts obtained with the same procedures used for ventricular trabeculae described previously [20]. 30 consecutive APs for each fibre were recorded at 1 and 2 Hz under control conditions (DMSO 0.1%), and with 100 nM dofetilide.

As explained below, Datasets I and II were used for model development and calibration, while Dataset III was used exclusively for model validation.

### 2.2 Strategy for model design, calibration and validation

**Fig. 1:**
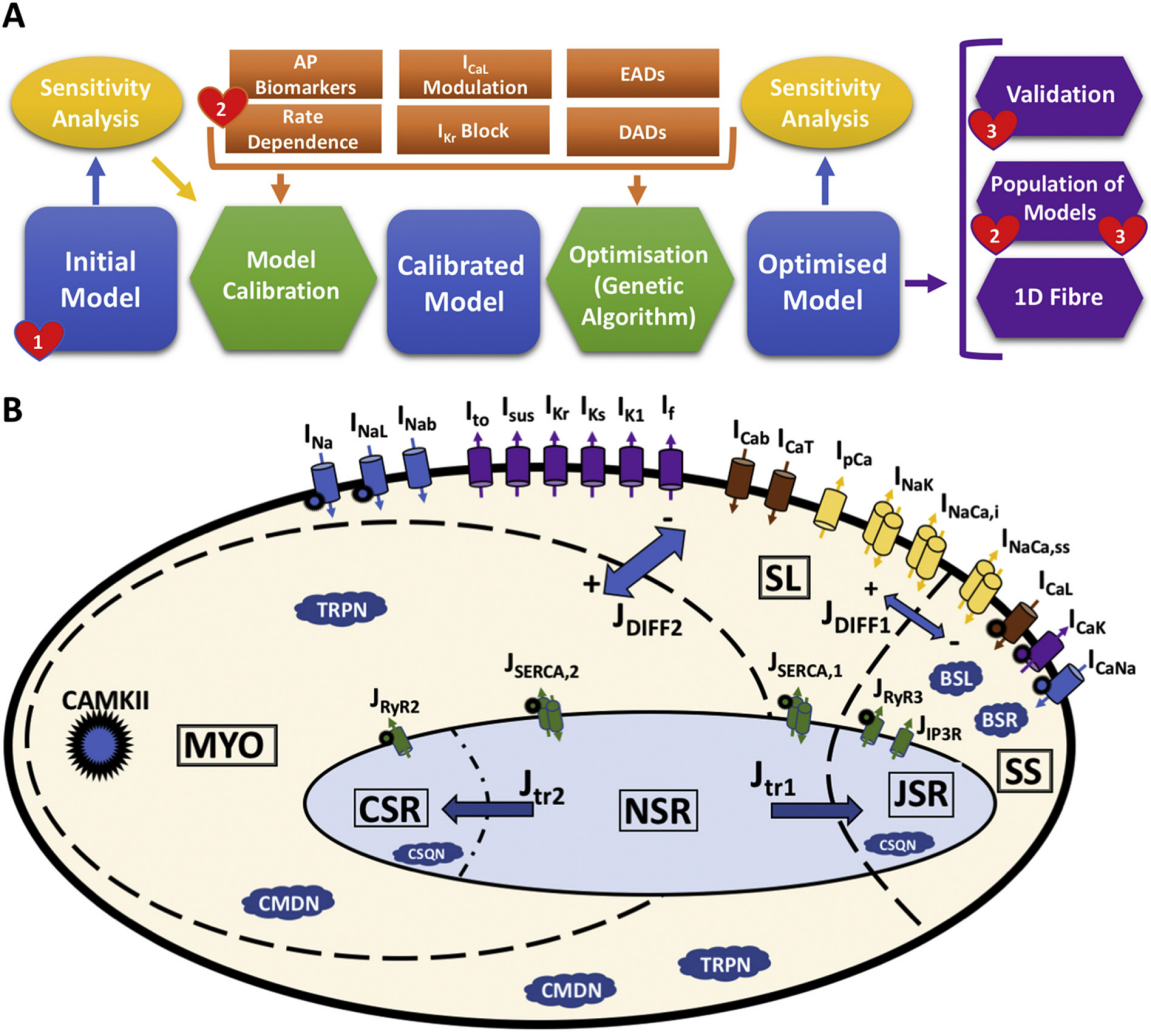
A) Schematic representation of the human Purkinje model development strategy. Blue: model development stages; green: data processing for calibration and optimisation; yellow: sensitivity analysis; orange: criteria for model development; purple: model validation and applications; red hearts: experimental datasets. B) Main structure and ionic currents of the Trovato2020 Purkinje AP model. The intracellular space is represented with 3 different compartments: peripheral coupling subspace (SS), sub-sarcolemma (SL), and bulk myoplasm (MYO). The sarcoplasmic reticulum (SR) also consists of 3 compartments: junctional (JSR), network (NSR), and corbular (CSR). 18 dynamic current models are included for Na^+^ (blue), K^+^ (purple) and Ca^2+^ (brown) channels, Na^+^-K^+^ pump, and Na^+^-Ca^2+^ exchanger (yellow). Intracellular Ca^2+^ release and up-take fluxes (green) are distributed across the 3 SR compartments. Ca^2+^ buffers are shown as blue clouds. Global CaMKII phosphorylation is also included, and the affected currents are marked by a spiky circle. (For interpretation of the references to colour in this figure legend, the reader is referred to the web version of this article.)

Fig. 1A illustrates the design, calibration, optimisation, and validation of the Trovato2020 model, described in more detail below and in the Supplementary Material, Section 1. The Trovato2020 model presented in this study was built based on the ionic formulations of the O'Hara-Rudy human ventricular model, ORd [21], and the Purkinje-specific Ca^2+^ sub-system, cellular compartments and intracellular ionic fluxes of the canine Purkinje AP model, PRd [14], also used by Britton et al. [19]. Fig. 1B reports a diagrammatic representation of the model structure. In brief, the Trovato2020 model includes the ORd mathematical formulation for each of the following currents: fast Na^+^ current (I_Na_), Na^+^ late component (I_NaL_), L-type Ca^2+^ current (I_CaL_), rapid and slow delayed K^+^ rectifiers (I_Kr_ and I_Ks_, respectively), Na^+^-Ca^2+^ exchanger (I_NCX_) and Na^+^-K^+^ pump (I_NaK_). I_to_, I_sus_ and I_K1_ were formulated based on the Dataset I. In addition, two Purkinje-specific currents from the PRd model were included: T-type Ca^2+^ current (I_CaT_) and funny current (I_f_). Existing knowledge from the literature on the ionic current differences between ventricular and PCs, and between human and canine PCs, was also taken into account.

The Trovato2020 model was developed to fulfil the following 6 criteria:

1) Consistency with the experimental AP biomarkers at all BCLs: simulated values had to be within the experimental ranges shown in Table 1.
2) APD_90_ rate-dependence: APD_90_ increase for BCLs between 400 ms and 2000 ms.
3) APD_90_ changes induced by I_CaL_ modulation: APD_90_ increase/shortening in response to I_CaL_ up/down regulation. The effect of I_CaL_ on APD_90_ has been shown experimentally, using selective Ca^2+^ blockers such as diltiazem [22] and nifedipine [23], i.e., I_CaL_ reduction induces AP shortening, whereas I_CaL_ increase leads to AP prolongation. Due to the lack of quantitative information and human data, we only imposed a positive correlation between changes in APD_90_ (∆APD_90_) and I_CaL_ up/down regulation (|∆APD_90_| > 0.5% with ±30% I_CaL_).
4) APD_90_ prolongation induced by I_Kr_ block. Experimental studies by [24] showed I_Kr_ block led to a longer APD in Purkinje than in ventricular cardiomyocytes. Thus, during calibration, we imposed the APD using the Trovato2020 model to be longer than the one obtained using the ventricular ORd model for both 30% and 50% I_Kr_ block.
5) Ability to generate EADs, reported experimentally in canine PCs [25,26].
6) Ability to generate DADs and/or triggered APs, as reported for canine PCs [27,28].

Starting from the initial model described above, a sensitivity analysis was performed to investigate how properties relevant to the 6 criteria were affected by variations in the ionic current parameters, and to guide the definition of a calibrated model (details in the Supplementary material, Section 1 and Fig. S2-S3). Parameter optimisation was then performed using a multi-objective genetic algorithm [29]. The conductances of the main currents, namely I_Na_, I_NaL_, I_CaL_, I_CaT_, I_to_, I_sus_, I_Kr_, I_Ks_, I_f_, I_K1_, I_NCX_, I_NaK_, as well as 7 parameters for I_CaL_ and I_Kr_ kinetics were allowed to vary in the ranges reported in Table S1, building on the sensitivity analysis results. The algorithm was run for 30 generations, with 300 models each. The multi-object cost function was computed as a weighted sum of 2 error functions accounting for the criteria listed above (details in the Supplementary Material, Section S1.3, Table S1).

The optimised Trovato2020 model was evaluated against Dataset III, and on its ability to develop automaticity under specific conditions (details in Section 2.3).

A population of models was generated using the optimised Trovato2020 model as baseline, to reproduce the experimental variability observed in Dataset II, and to evaluate model stability to parameter variations (details in Section 2.4). Propagation of electrical excitation in a 1D fibre using the optimised Trovato2020 model was also simulated, to verify conduction velocity (CV) in control conditions, as well as to test the potential propagation of spontaneous APs, EADs and DADs in tissue, and to evaluate the effects of I_Na_ block on CV (details in the Supplementary Material, Section 1.4).

### 2.3 Model validation

The optimised model was evaluated against the experimental Dataset III, not considered during the model development and not used for the calibration and optimisation of the initial model. The simulations in control conditions and with dofetilide were conducted in similar experimental conditions as for Dataset III, following the protocols 1 and 7 (Section 2.5), respectively.

Moreover, the potential for automaticity was investigated using the protocol outlined below (Section 2.5), by modifying the balance between I_K1_ and I_f_, which determined the DMP [2,7]. Furthermore, the effect of hyperkalaemia was also investigated by increasing the extracellular potassium concentration, since it has been shown to suppress automaticity in human PCs [8].

### 2.4 Population of models

Using the optimised model as baseline, we constructed a population of human Purkinje models, based on the methodology described in [18,19]. This allowed to simulate the variability in AP morphology observed in the experimental recordings (Table 1).

An initial population of 3000 models was constructed by sampling all the main ionic current conductances, namely, I_Na_, I_NaL_, I_CaL_, I_CaT_, I_to_, I_sus_, I_Kr_, I_Ks_, I_f_, I_K1_, I_NCX_, I_NaK_, in the range [50–200]% of their baseline value, using Latin hypercube sampling [30]. The initial population was then calibrated through a multi-step process, based on the criteria 1–3 used for the development of the Trovato2020 model and our human experimental Datasets II and III.

In the first calibration step, only the models with all AP biomarkers within the minimum and maximum experimental values at all BCLs (Table 1) were selected. To constrain the AP plateau within the experimental traces, two additional biomarkers at 1 Hz were considered, similar to [19]: the voltage level measured 25 and 50 ms after the AP upstroke. In the second calibration step we selected only the models showing APD_90_ rate-dependence in line with experiments. As third calibration step, we selected only the models showing a direct dependence of APD_90_ on I_CaL_.

Simulations in control conditions and with dofetilide were performed for all models in the calibrated population, and results were compared to the experimental Dataset III. Finally, EADs and DADs inducibility was also tested using the calibrated population, to investigate the mechanisms underlying EADs and DADs generation in human PCs. To do this, protocols 5 and 6 (Section 2.5) were simulated for each model in the calibrated population. EADs were identified when a positive derivative of the membrane potential was observed, from 150 ms after the stimulus [31]. DADs were identified as a deflection of the membrane potential larger than 1 mV during the diastolic phase. AP biomarkers, current conductances and model dynamics were analysed to identify the key mechanisms underlying EADs and DADs generation.

### 2.5 Stimulation protocols

Single cell model equations were implemented in Matlab (Mathworks Inc. Natwick, MA, USA) and solved with the function *ode15s*, an adaptive time step solver for stiff problems [32]. Data analysis was also performed using Matlab. Simulations for the population of models were run on the Oxford supercomputer ARCUS (http://www.arc.ox.ac.uk/). The monodomain formulation was used to simulate propagation along the fibre [33] and was solved using the Fourier spectral method for fractional diffusion [34]. The Rush-Larsen method was implemented for the integration to speed up the simulations [35] in Matlab.

The calibration and validation criteria described in Section 2.2 were evaluated using 9 different simulation protocols, 6 for calibration, 2 for validation and 1 for comparison against voltage-clamp experiments. A list of the protocols is provided below:

1) Control conditions. Steady state (SS): 1000 beats at 1 Hz, to allow the intracellular concentrations to reach stability.
2) Rate dependence. Starting from SS, 150 beats for each BCL, from 300 ms to 5000 ms.
3) I_CaL_ modulation. Starting from SS, 150 beats at 1 Hz with up/down regulation of the I_CaL_ conductance (±30%).
4) I_Kr_ block. Starting from SS, 150 beats at 1 Hz with I_Kr_ block (at 30% and 50%).
5) EADs inducibility. Starting from SS, 150 beats at slow pacing (BCL = 4000 ms) with 85% I_Kr_ block, as in [21].
6) DADs inducibility. Starting from SS, 1500 beats at fast pacing (BCL = 300 ms, 3.3 Hz), with and without RyR hypersensitivity. The model was then left unstimulated for 10s to allow for any potential DADs or triggered APs to arise. RyR hypersensitivity was simulated as an increased sensitivity to intracellular Ca^2+^ (+100%) and a decrease in the release time constant (−70%), similar to [14].
7) Dofetilide. Starting from SS, 150 beats at 1 Hz and 2 Hz, using a simple pore-block drug model [36] with IC_50_ (in μM) and Hill coefficient (IC_50_/h) for I_Kr_, I_Na_ and I_CaL_ equal to 0.03/1.2, 162.1/1 and 26.7/1, respectively [37].
8) Automaticity. Starting from SS, 3 different combinations of I_f_ and I_K1_ were tested: i) 90% I_K1_ block; ii) increasing I_f_ 9-folds; iii) combining the 2 conditions, 50% I_K1_ block and 4-folds I_f_. The values of I_K1_ and I_f_ conductances were identified through a sensitivity analysis up to automaticity appearance. The model was stimulated for 150 beats at 1 Hz, followed by 15 s with no stimulation. The same simulations were repeated under hyperkalaemia with extracellular [K^+^] set to 8 mM to reproduce the same conditions as in [8].
9) Voltage clamp simulations (for I-V curves). 1000 ms at holding voltage (−40 for I_K1_ and − 50 mV for both I_to_ and I_sus_) followed by a step (300 ms for I_to_ and 100 ms for both I_K1_ and I_sus_) at different voltage values (from −120 mV to 0 mV for I_K1_, and from −50 mV to +60 mV for both I_to_ and I_sus_). Intracellular [Na^+^], [Ca^2+^] and [K^+^] were set to 0.0001 mM, 0.0001 mM and 130 mM, respectively, while their corresponding extracellular concentrations were 140 mM, 1 mM, 5.4 mM, respectively, as in the experiments by Han et al. [9].

## 3 Results

### 3.1 Simulations with the optimised Trovato2020 model reproduce experimental AP recordings and fulfil all calibration criteria

Optimisation using the genetic algorithm produced a Pareto optimal front of 105 models, including many duplicates of 8 unique models. The final optimised Trovato2020 model was identified as the model with the best performance across all the tested protocols. Parameters and simulation results for the 8 models are shown in Fig. S4. Table 2 reports the simulated AP biomarkers for the optimised Trovato2020 vs the non-optimised model, and the human experimental biomarker ranges from Dataset II at 1 Hz.

**Table 2:** Simulated and experimental AP biomarkers.

	Experiments	Initial model	Optimised model
dV/dt_Max_ (V/s)	387 ± 143	435	381
APD_90_ (ms)	294 ± 76	249	306
APD_75_ (ms)	261 ± 67	225	279
APD_50_ (ms)	210 ± 52	168	223
APD_25_ (ms)	117 ± 46	116	142
APD_10_ (ms)	33 ± 35	49	34
APA (mV)	106 ± 7	113	110
TOP (mV)	−85 ± 2.4	−86.7	−86.5
EOP (mV)	−86 ± 2	−87.2	−87.3

Comparison between experimental and simulated AP biomarkers using the initial and optimised models at 1 Hz (mean ± std).

**dV/dt**_**MAX**_**:** maximum upstroke velocity; **APD**_**x**_**:** AP duration at X% of repolarisation; **APA:** action potential amplitude; **TOP**: membrane potential before depolarisation; **EOP:** membrane potential at the end of repolarisation.

**Fig. 2:**
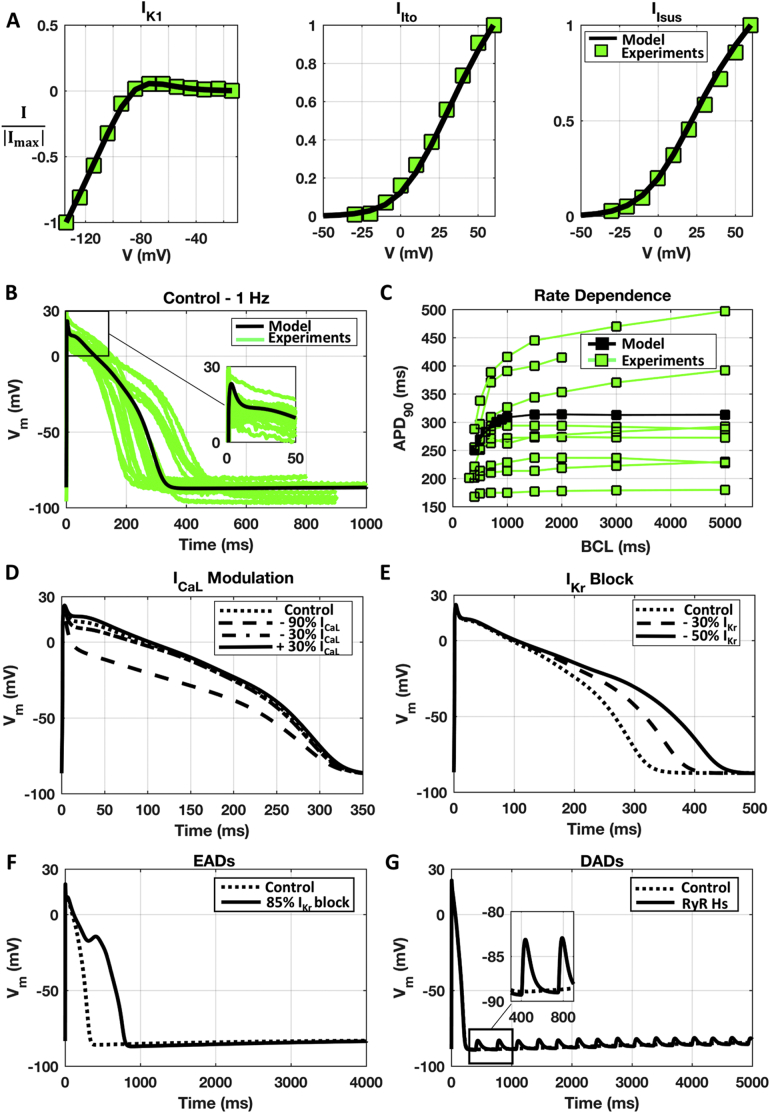
Simulations results obtained with the optimised Trovato2020 model (black), compared against the corresponding experimental data (green), when available: A) I-V curves for I_K1_ (left), I_to_ (middle) and I_sus_ (right). Experimental data from Dataset I [9]. B) AP traces at 1 Hz. Experimental data from Dataset II. C) APD_90_ rate-dependence. Experimental data from Dataset II. D) AP changes induced by I_CaL_ conductance modulation. E) AP changes induced by I_Kr_ block. F) EADs observed at slow pacing with I_Kr_ block. G) DADs following fast pacing with RyR hypersensitivity. No DADs were observed in control. (For interpretation of the references to colour in this figure legend, the reader is referred to the web version of this article.)

Fig. 2 shows the simulation results for the optimised Trovato2020 model, reproducing Datasets I and II and satisfying each of the 6 calibration criteria: simulations of voltage-clamp protocols for I_to_, I_sus_ and I_K1_ to reproduce the experimental I–V curves from Dataset I (Panel A); AP traces at 1 Hz and rate dependence in line with experimental recordings from Dataset II (Panel B and C, respectively); APD_90_ changes induced by I_CaL_ modulation (Panel D) and I_Kr_ block (Panel E); EADs and DADs inducibility (Panel F and G, respectively). Further information on the ionic currents underlying the AP and Ca^2+^-transient in the optimised model at 1 Hz are included in the Supplementary Material, Fig. S5.

As expected, fast Na^+^ current activation drives the depolarisation phase with dV/dt_MAX_ = 381 mV/s, 24 mV voltage peak and 110 mV amplitude (Table 2). During the early-repolarisation phase, simulated AP (Fig. 2B) presents a “spike and dome” waveform (Fig. 2B inset), due to the interplay between the depolarising currents I_CaL_ and I_NaL_ and the repolarising I_to_ and I_sus_. The repolarisation phase is driven by I_Kr_, I_Ks_ and I_K1_, resulting in APD_90_ of 306 ms at 1 Hz. The small diastolic depolarisation (~1 mV), due to the action of I_f_, is in agreement with the difference observed experimentally between potentials at take-off and end of repolarization (EOP vs TOP). No automaticity was observed, in agreement with experimental recordings from Dataset II and the literature [7]. The correlation coefficients between AP biomarkers and ionic current conductance at 1 Hz are reported in Table S2.

Rate-dependence of APD_90_ is in line with experimental recordings from Dataset II (Fig. 2C). At BCL = 400 ms, the APD_90_ is 250 ms and increases up to 312 ms at BCL = 5000 ms. Furthermore, the optimised model reproduces a positive correlation between APD and I_CaL_ modulation (Fig. 2D): small changes in I_CaL_ conductance affect the plateau phase, with small changes in APD_90_ as reported for rabbit PCs [38]. However, a stronger I_CaL_ block also causes APD_90_ shortening, as in experimental recordings using canine PCs [[22], [23]].

Simulations with the new human Purkinje Trovato2020 model yield APD prolongation following I_Kr_ block (Fig. 2E): 30% and 50% I_Kr_ blocks prolong APD_90_ from 306 (control) to 365 and 421 ms, respectively, whereas the same degrees of block in the human ventricular ORd model prolong the APD_90_ from 269 ms (control) to 329 and 386 ms, respectively. These results are in agreement with canine experiments [24] showing a longer AP in Purkinje compared to ventricular myocytes, both in control conditions and following I_Kr_ block.

EADs inducibility is shown in Fig. 2F: at BCL = 4000 ms, the AP fully repolarises in control (APD_90_ = 307 ms, dashed line), whereas an EAD occurs with 85% I_Kr_ block (solid line).

DADs inducibility is illustrated in Fig. 2G: following fast pacing, the Trovato2020 model remains well polarised in control conditions (dashed line), while DADs occur when including RyR hypersensitivity (solid line). The initial DADs amplitude is 6 mV, and it decreases to less than 1 mV after 13 s. Even after 300 s, the model exhibits oscillations albeit of amplitude smaller than 0.01 mV, and therefore potentially undetectable experimentally. The ionic mechanisms of EADs and DADs generation are investigated in Section 3.6 and 3.7.

Further analysis of the optimised Trovato2020 model is included in the Supplementary Material, including the sensitivity analysis (Section 4, Fig. SA1–SA5).

### 3.2 Model validation: response to dofetilide and automaticity

**Fig. 3:**
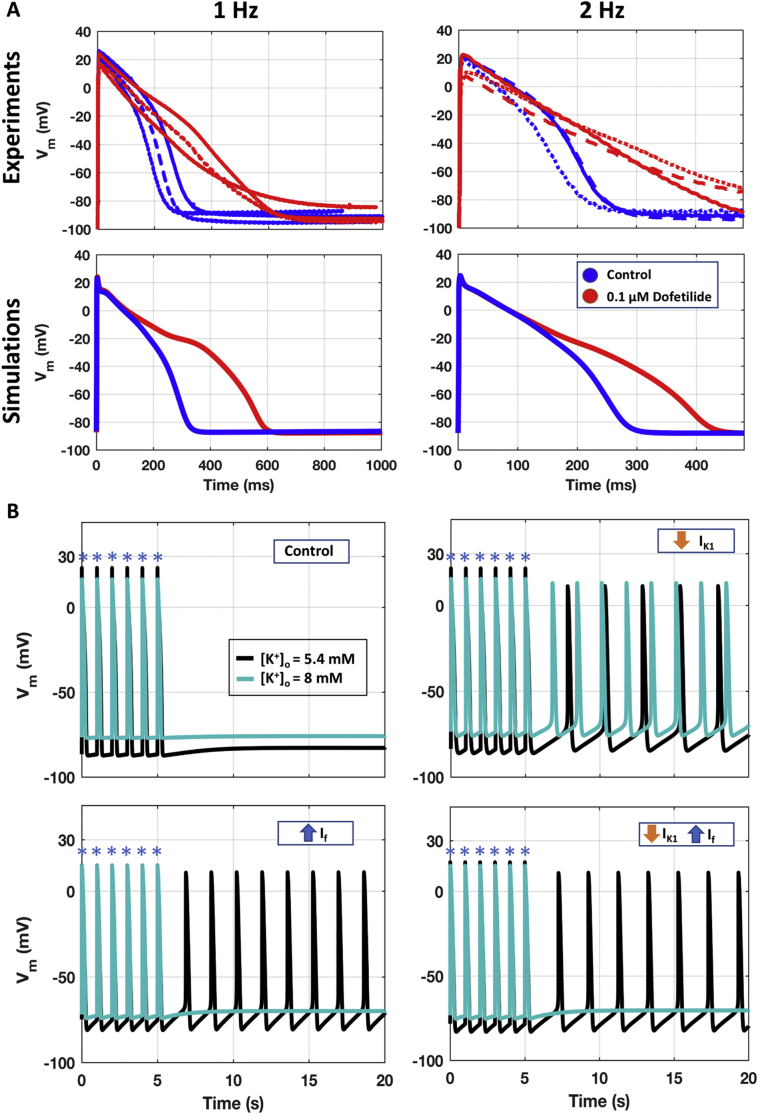
Independent validation of the optimised Trovato2020. A) Comparison between the human experimental AP traces from Dataset III (top panels, *n* = 3) and the simulations with the Trovato2020 model (bottom panels), in control conditions (blue) and with Dofetilide 100 nM (red), at 1 Hz (left) and 2 Hz (right). B) Ability of the model to develop automaticity when no external stimulus (blue asterisks) is applied. Simulations are shown both with normal [K^+^]_o_ (black) and under hyperkalaemia conditions (light blue), in four different scenarios. (For interpretation of the references to colour in this figure legend, the reader is referred to the web version of this article.)

Fig. 3A illustrates the agreement of simulation results with experiments from Dataset III, in control conditions (blue) and with dofetilide (red), at 1 and 2 Hz (left and right, respectively).

Fig. 3B illustrates the ability of the Trovato2020 model to display automaticity following I_K1_ decrease and/or I_f_ increase. When including either I_K1_ down-regulation, I_f_ up-regulation, or a combination of both, the DMP increases much faster during the resting phase, and automaticity is observed at BCL of 2.5, 1.6 and 1.9 s, respectively. These results are in line with the BCL range of 1.3–3.0 s experimentally observed by [8,15], and also with [2,39], reporting that PCs with less negative DMP show automaticity, while well polarised PCs remain quiescent. Hyperkalaemia stops the automaticity in the two scenarios including I_f_ up-regulation, in agreement with experiments in human PCs [8], whereas it increases the spontaneous firing rate when automaticity is induced only by I_K1_ down-regulation.

### 3.3 Comparison with previous cardiac Purkinje in silico models

The Trovato2020 model was compared with two other human Purkinje models available in the literature (TT08 and STW). Fig. S6A depicts the simulated APs at 1 Hz for the three models with experimental APs from Dataset II, while the corresponding AP biomarkers are reported in Table S3. Simulation results obtained with both TT08 and STW overestimate the rate of depolarisation (742 and 522 V/s, respectively), compared to experiments from Datasets II and III, and the literature, i.e., 388 ± 25 V/s [40] and 207 ± 26 V/s [8].

Trovato2020 and TT08 have similar DMP, whereas STW is significantly less negative. Indeed, STW shows automaticity in control conditions, while Trovato2020 reproduces the quiescent PCs in control, and automaticity with less negative DMP. TT08 does not include any formulation for the I_f_, and it does not yield automaticity under the protocols considered in this study (Section 2.5).

Both Trovato2020 and TT08 show an APD rate dependence in line with the experiments (Fig. S6B), even if TT08 largely overestimates the APD_90_ at all BCL, while STW shows a non-physiological rate dependence. Simulations with TT08 and STW failed to reproduce EADs with protocol 5 (Fig. S6C), only showing AP prolongation (17% and 42%, respectively). Both TT08 and STW also underestimate the AP prolongation induced by dofetilide (Fig. S6D), compared to our Dataset III. Finally, neither TT08 nor STW are capable of developing DADs at fast pacing, even when considering RyR hypersensitivity. Therefore, the novel Trovato2020 model is more suitable than both TT08 and STW for mechanistic investigations of arrhythmias considering EADs, DADs, triggered activity as well as APD rate dependence.

The Trovato2020 model was also compared with the ORd and PRd models: simulated AP biomarkers are reported in Table S3, whereas, APs, intracellular Ca^2+^ concentrations and the three refitted K^+^ currents, I_to_, I_sus_, I_K1_, at 1 Hz are reported in Fig. S6E. The simulated AP with the Trovato2020 model displays a smaller peak potential than both ORd and PRd, faster upstroke velocity than ORd but slower than PRd, AP plateau similar to PRd and lower than ORd, and finally, APD_90_ longer than ORd but shorter than PRd. Intracellular [Ca^2+^] is smaller with the Trovato2020 model than with ORd, but similar to the one with PRd, which was based on experimental data from canine PCs [41]. In particular, Trovato2020 and PRd have the same level of diastolic [Ca^2+^] and same delay in the Ca elevation time (~100 ms). Similarly, the [Ca^2+^] of the sub membrane compartment obtained with the Trovato2020 was similar to the one simulated with PRd, whereas the ORd does not implement such compartment.

The refitted K^+^ currents I_to_, I_sus_ and I_K1_ were compared as well (Fig. S6E). The simulated I_to_ with Trovato2020 is bigger than with ORd but smaller than PRd, as suggested by experiments in human and canine PCs [[9], [42]]. In all three models, its contribution ends after 20 ms. I_sus_, not implemented in ORd, displays lower amplitude in Trovato2020 than in PRd, similarly to I_to_. Simulated I_K1_ with Trovato2020 is smaller than both ORd and PRd, in line with the reduced level of expression of I_K1_ proteins in human Purkinje [43,44].

Trovato2020, ORd and PRd share the same model for CaMKII signalling [45]. Similarly to the ORd, removing CaMKII from the Trovato2020 model (Fig. S7A) reduces intracellular and submembrane [Ca^2+^] peak, with minimum changes on the APD and [Ca^2+^] rate-dependence. The decreased [Ca^2+^] due to CaMKII signalling removal had no effects on DADs induced at fast pacing with RyR hypersensitivity (Fig. S7B), though also the sarcoplasmic [Ca^2+^] was reduced. No effects were observed on EADs dynamics, as expected due to the low pacing frequency.

### 3.4 A population of human Purkinje Trovato2020 models accounting for biological variability

**Fig. 4:**
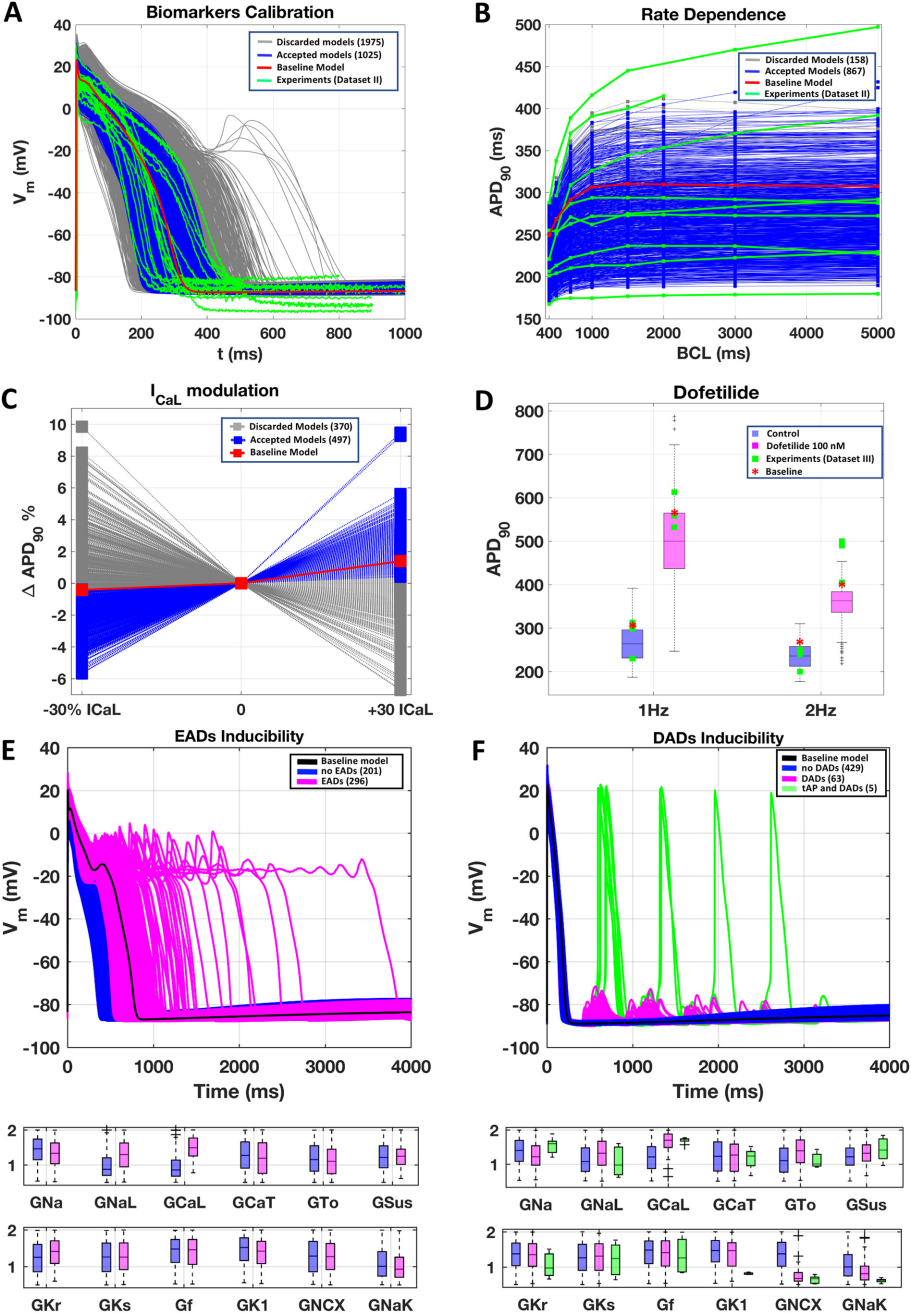
Simulation results for the population of Trovato2020 models, including calibration (A-C), validation (D), and investigation of EAD and DAD inducibility (E-F). A) Results of the first calibration step, based on AP biomarkers. AP traces are shown for the baseline model (red), the discarded models (grey), and the accepted models (blue), compared against experimental traces from Dataset II (green). B) Results of the second calibration step, based on APD_90_ rate-dependence. C) Results of the third calibration step, based on I_CaL_ modulation of APD_90_. D) Comparison of APs with the experimentally-calibrated population of Trovato2020 models against experimental data from Dataset III. Boxplots represent the APD_90_ distribution at 1 Hz (left) and 2 Hz (right) in control conditions (blue) and with Dofetilide 100 nM (pink). Simulation results with the baseline model are reported as a red star, while experimental data are shown as green squares. On each box, the central mark is the median of the population, box limits are the 25th and 75th percentiles, and whiskers extend to the most extreme data points not considered outliers, plotted individually as separate crosses. E) Top panel: Simulated AP traces for the EADs protocol, including the baseline model (black), models not displaying EADs (blue), and models displaying EADs (pink). The baseline model is shown in black. Bottom panel: Distribution of the scaling factors of the ionic current conductances varied in the population of models, highlighting the differences between the two groups of models. F) Top panel: Simulated AP traces for the DADs protocol, including the baseline model (black), models not displaying DADs (blue), models displaying DADs (pink), and models displaying triggered APs (green). Bottom panel: distribution of the scaling factors of the ionic current conductances varied in the population, highlighting the differences between the three groups of models. Boxplot description as in D. (For interpretation of the references to colour in this figure legend, the reader is referred to the web version of this article.)

The optimised human Trovato2020 model was used as baseline to construct a population of models, to capture the biological variability in the AP morphology observed in the experimental Dataset II. The initial population (*n* = 3000) was calibrated through a multi-step filtering process, as described in Methods, and summarised in Fig. 4.

In the first calibration step, only the models in agreement with the experimental biomarkers at all BCLs were selected (*n* = 1025), while the others were discarded (*n* = 1975). Fig. 4A reports the simulated APs for the baseline Trovato2020 model, the initial population and the experimental traces at 1 Hz. In the second calibration step, *n* = 867 models showed APD_90_ rate dependence in line with the experiments and were kept in the population (Fig. 4B). In the last calibration step, *n* = 497 models were accepted into the final population, all displaying a positive correlation between APD and I_CaL_ (Fig. 4C). These models were used for all subsequent investigations.

Fig. 4D illustrates the APD distribution in the population, both in control conditions (blue) and with dofetilide (pink), at 1 Hz and 2 Hz (left and right, respectively), compared against the experimental APD values from Dataset III. Simulation results for the population are in agreement with the experimental values in control conditions and with dofetilide at 1 Hz. The simulated APs with the population yield a wider range of APD prolongation compared to experiments, similar to what was previously shown for rabbit PCs [19].

### 3.5 EADs mechanisms

**Fig. 5:**
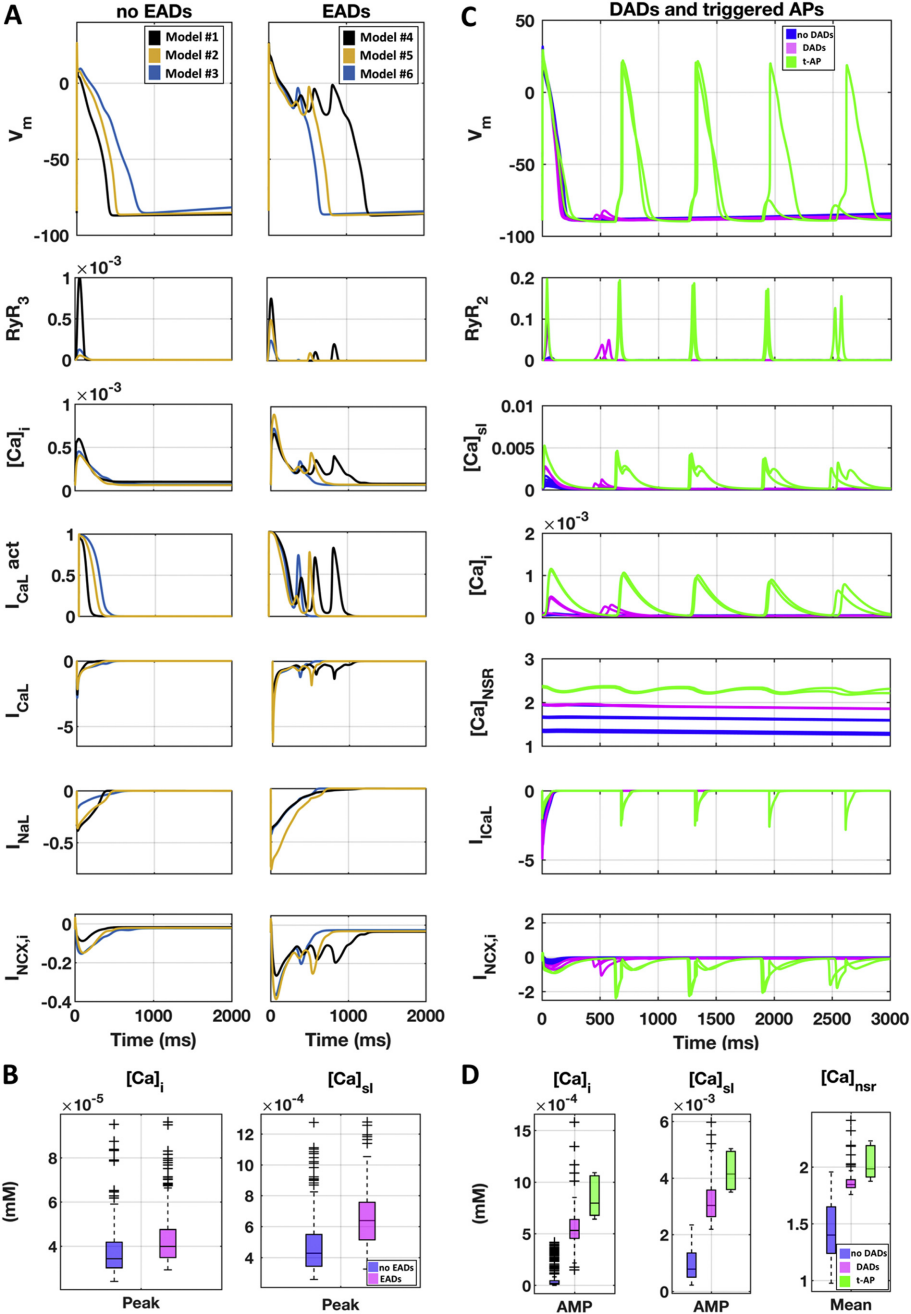
EADs and DADs mechanisms. A) Simulations of the EADs protocol, showing the AP and the main currents/concentrations involved in EADs generation. 6 representative models are shown: 3 not displaying EADs (left), and 3 displaying EADs (right). All models exhibiting EADs display I_CaL_ re-activation, as shown by I_CaL_ activation gate dynamics (4th row from top). B) Intracellular and sub membrane [Ca^2+^] peaks (computed before I_Kr_ block) for models showing only AP prolongation (blue) and those developing (EADs). C) Simulated APs and main currents/concentrations involved in DADs generation. 17 representative models are shown: 10 not displaying DADs (blue), 5 displaying DADs (pink), and 2 developing triggered APs (green). All models displaying DADs or triggered APs have higher [Ca^2+^]_i_, [Ca^2+^]_sl_ and [Ca^2+^]_NSR_. D) Intracellular and sub membrane [Ca^2+^] amplitude, and mean sarcoplasmic [Ca^2+^] for all the models in the calibrated population: not displaying DADs (blue), displaying DADs (pink) and developing triggered APs (green). (For interpretation of the references to colour in this figure legend, the reader is referred to the web version of this article.)

When simulating the EADs protocol (described in Section 2.5) in the experimentally-calibrated population of models (Fig. 4E, top panel), 59% of the models (*n* = 296) developed EADs (pink traces), while the rest of the models only displayed AP prolongation (blue traces). Multiple EAD phenotypes were observed, in agreement with previous experimental and simulation studies [31,46,47]. The distribution of the ionic current conductances (Fig. 4E, bottom panel) highlights the differences between these two groups of models. Models displaying EADs were mainly characterised by larger inward current conductances (G_CaL_ and G_NaL_), in agreement with the sensitivity analysis results (Supplemental Material, Section 4, Figure SA4). I_CaL_ re-activation (Fig. 5A) was identified as the key mechanism for EAD generation [48,49] whereas, no I_NaL_ reactivation was observed with the protocol used in this study [50]. Fig. 5B reports intracellular and sub membrane [Ca^2+^] peak values in control using the models displaying only AP prolongation and those developing EADs.

### 3.6 DADs mechanisms

When simulating the DADs protocol (described in Section 2.5) in the experimentally-calibrated population (Fig. 4F, top panel), 63 models developed DADs (pink traces), 5 models developed triggered APs (green traces), while no DADs were observed in the remaining models (blue traces). The distribution of the ionic current conductances (Fig. 4F, bottom panel) highlighted the differences between these three groups of Purkinje models. Human virtual PCs displaying DADs had larger G_CaL_, and reduced G_NCX_.

Fig. 5C illustrates the ionic mechanisms underlying DADs formation. Models displaying DADs showed higher Ca^2+^ concentrations in all intracellular compartments, including the SR. In particular, in all models displaying DADs, [Ca^2+^]_NSR_ was larger than 1.7 mM, in line with previous computational studies [[51], [52], [53]] and also larger than in the baseline model with RyR hypersensitivity (1.1 mM, Fig. S7B). Ca^2+^ accumulation made the cell more vulnerable to spontaneous SR Ca^2+^ release, which were translated by the I_NCX_ into the depolarising currents causing DADs. Fig. 5D shows intracellular and submembrane [Ca^2+^] amplitudes, i.e., the difference between the diastolic concentration and the peak value for each cellular compartment, and the average sarcoplasmic [Ca^2+^] of the models staying quiescent and those developing DADs and triggered APs across the whole calibrated population.

In order to establish the link between the distribution of ionic current conductances and Ca^2+^ accumulation in the models displaying DADs, we performed additional ad hoc simulations for a selection of 3 models from the population. We selectively restored ionic current conductances to their baseline values, and evaluated changes in Ca^2+^ accumulation and DADs generation. Large G_CaL_ and small G_NCX_ directly contributed to Ca^2+^ overload and DADs generation: restoring the value of either of these conductances to their baseline significantly reduced intracellular Ca^2+^ concentrations, and also abolished DADs (Fig. S8, Panel A and B).

Triggered APs were observed in ~1% of the experimentally-calibrated population (*n* = 5 models). All models developing triggered APs were characterised by smaller G_K1_ and G_NaK_ (Fig. 4F), both reducing the outward current during the diastolic phase, and had higher intracellular Ca^2+^ concentrations, compared to the ones developing DADs only (Figure 5B).

### 3.7 Simulation results in a human Purkinje 1D fibre

**Fig. 6:**
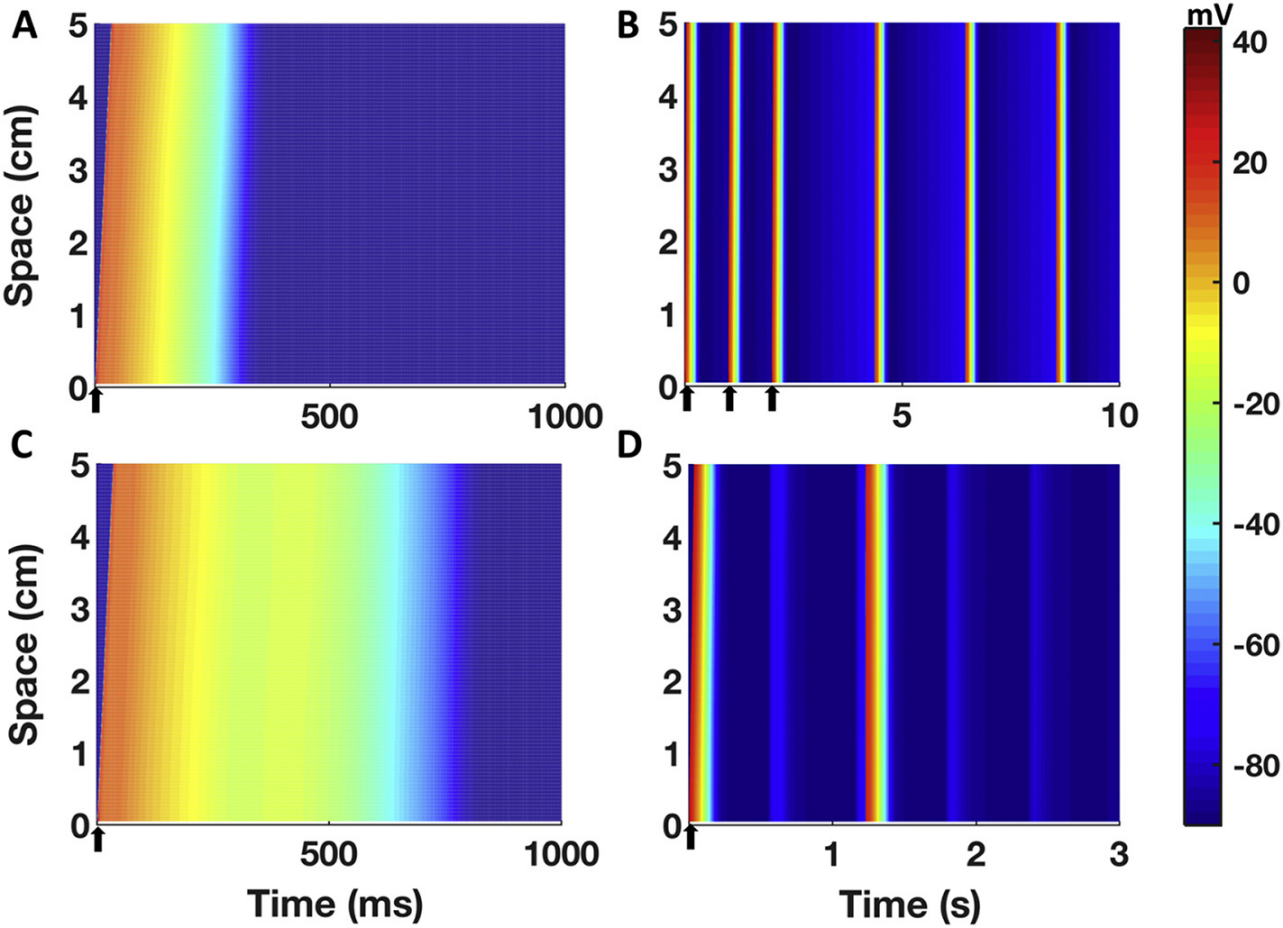
Simulation results for a 1D human cardiac Purkinje fibre constructed using the Trovato2020 model. **A)** Simulated AP in control conditions, propagating along the fibre. The delay between the AP upstroke at the proximal and distal ends is 32 ms. B) Simulation results obtained with one of the variants of the Trovato2020 model displaying automaticity (increased I_f_, reduced I_K1_). Spontaneous APs were observed in the fibre, and the CL was not different than the one obtained in single cell simulations. **C)** Simulation results for the EADs inducibility protocol. EADs propagated along the fibre. **D)** Simulation results for the DADs inducibility protocol, using one of the models in the population displaying triggered APs. The cellular coupling reduced the number of triggered APs compared to single cell simulations (from 2 to 1). All the panels share the same colour map, displayed on the right. Please note the different time scales (ms for A and C, s for B and D). Arrows indicate time and sites of pacing.

Fig. 6 summarises the simulations results for the human Purkinje 1D fibre considering the optimised Trovato2020 model in control conditions (Fig. 6A) and one of the model variants displaying automaticity in single cell as reported in Section 3.2 (Fig. 6B). Spontaneous APs propagated along the whole fibre, with no changes in the BCL compared to single cell simulations. Fig. 6C shows the results with the optimised Trovato2020 model and the EADs protocol: EADs were observed in the whole fibre, with no changes in EADs amplitude induced by the intercellular coupling. Fig. 6D shows the results of one of the models in the population developing both triggered APs and DADs at fast pacing (Fig. 5C): in this case, the electrical coupling affected the simulations since only a single triggered APs was observed in fibre, compared to the 2 observed in the single cell simulations. After the fast pacing, the fibre depolarised generating one DAD (*t* = 550 ms, Fig. 6D) but it did not reach the threshold to allow fast Na^+^ channels opening. Though, after another further 650 ms the membrane reached the threshold and a spontaneous AP was elicited, followed by 2 DADs, as in the single cell simulations. However, when the electrophysiological changes underlying EADs and DADs were applied only to the central portion of the fibre (1.67 cm, 33 nodes), abnormalities did not propagate due to the sink-source mismatch as previously reported by Xie et al. [54].

I_Na_ block caused reduction in CV (Table S4): for I_Na_ blocks of 30%, 50% and 90%, CV decreased by 8%, 15% and 45%, respectively, compared to the control value of 160 cm/s. Even with 95% I_Na_ block, the AP was still able to propagate, even though CV was reduced to 69 cm/s (−57%). AP failed to propagate only with a complete block of I_Na_.

## 4 Discussion

### 4.1 The novel human Purkinje AP model

In this study, the novel human PC Trovato2020 model was presented, including its construction, calibration, optimisation and independent evaluation using experimental AP recordings from undiseased human PCs and knowledge about Purkinje-specific currents and Ca^2+^-handling. Parameter optimisation was performed using a multi-objective genetic algorithm and sensitivity analysis, to overcome manual tuning limitations. Independent model validation was conducted using AP recordings from undiseased human PCs in control and with dofetilide, and also based on the model's ability to reproduce EADs, DADs and trigger activity using specific protocols. Both single cell and 1D fibre simulations were performed using a variety of stimulation protocols. In addition, an experimentally-calibrated population of human PC models was also constructed to account for biological variability, and used to investigate the ionic mechanisms underlying EADs and DADs generation in human PCs. The main findings of this study are:

1. Simulation results with the Trovato2020 model are in agreement with the key features of human Purkinje APs reported in the human experimental datasets presented in this study and from the literature. Simulations reproduce a wide range of stimulation protocols, including different pacing frequencies and selective channel blocks.
2. The Trovato2020 model is able to yield and explain pro-arrhythmic mechanisms, i.e. EADs and DADs, in human PCs. Simulations show that 59% of the PCs models in the population display EADs caused by I_CaL_ reactivation and favoured by strong I_CaL_ and I_NaL_. DADs are observed in 13% of the Purkinje population models, displaying Ca^2+^ accumulation both in the intracellular space and into the sarcoplasmic reticulum. Triggered APs were observed in 1% of the virtual PCs, which displayed strong downregulation of I_K1_ and I_NaK_.
3. Integration of the Trovato2020 model in a 1D fibre succeed to reproduce AP propagation, automaticity, EADs, DADs and triggered APs in tissue. It can be implemented in higher scale models for investigation also at tissue and organ level.

The Trovato2020 Purkinje model integrates and expands a large amount of knowledge and experimental data obtained from human Purkinje preparations. It is the first human Purkinje model incorporating a Ca^2+^ handling with the 3 SR compartments and 3 different types of Ca^2+^ releases, based on the Purkinje-specific structure [55] already implemented for rabbit [19] and canine [14] models. Previously published human Purkinje models [[15], [16], [17]] include only 2 intracellular compartments and 1 type of Ca^2+^ release, inherited from ventricular models. Thus, they do not account for Purkinje specific features such as the low density of t-tubuli and different type of Ca^2+^ releases. A physiological Purkinje Ca^2+^-handling representation is crucial, since it can favour arrhythmogenesis in pathology [1] and upon pharmacology interventions [13]. The Purkinje-specific Ca^2+^ handling model introduces a delay in the Ca^2+^ diffusion from the sub-membrane to the cellular bulk, and therefore, an intracellular [Ca^2+^] gradient (Fig. S5). The simulated [Ca^2+^]_i_ of the new Purkinje model in control conditions is smaller and delayed compared to the [Ca^2+^] in the sub-membrane space as reported by [[55], [56], [57]]. The smaller Ca^2+^ transient is correlated with scarce myofibrils, according to the lower contractile ability of Purkinje compared to ventricular cardiomyocytes [13].

Simulations with the human Purkinje Trovato2020 model revealed that the balance between I_K1_ and I_f_ determines the DMP and, therefore also automaticity, as previously suggested experimentally by [2,58]. The simulation results are also in agreement with [8], in showing pacemaker activity in human PCs with elevated diastolic transmembrane potential (TOP ~ −70) in control condition. Hyperkalaemia stopped pacemaker activity, as also reported experimentally [8], but only when automaticity was due to I_f_ up-regulation, since high potassium concentration up-regulates I_K1_ making the resting more stable. This does not occur when automaticity was induced only by I_K1_ down-regulation, suggesting a more physiological role of I_f_ in setting PC automaticity compared to pure I_K1_ down-regulation’. Heterogeneity in the DMP and automaticity may lead to conduction block, creating the substrate for micro and/or macro re-entry, and eventually arrhythmia [1].

The human Purkinje Trovato2020 model was also able to reproduce the effects of potassium and calcium channel block on repolarization properties, as well as sodium channel block on electrical propagation. The simulation results were in agreement with human experimental data acquired at several frequencies, both in control conditions and following dofetilide application. Simulations with dofetilide at 2 Hz (Fig. 4D) showed reverse rate-dependence, i.e., less prolongation at higher frequencies, in agreement with observations both in human and animal preparations [59], while a larger APD prolongation is observed in the human recordings from Dataset III. These results lend credibility to the new model for in silico drug trials in human PCs to assess drug efficacy and/or drug-induced cardiotoxicity, as done using human ventricular models [60].

### 4.2 EADs and DADs mechanisms

Afterdepolarisations in Purkinje cardiomyocytes can act as triggers of ectopic activity and arrhythmia, particularly in diseased or drug action conditions [1]. The Trovato2020 model is the first human Purkinje model able to reproduce EADs, DADs and triggered AP in single cell and 1D fibre. This enables the investigation of PCs as arrhythmia triggers [61,62], in addition to their contribution to the substrate for re-entry circuits and retrograde propagation [63,64]. Therefore, the integration of the Trovato2020 model in tissue or whole-ventricular models, including the Purkinje tree [65,66], could enable investigations into the role of PCs in human ventricular arrhythmias, caused by relevant disease conditions such as myocardial infarction [[67], [68], [69], [70], [71]], ischemic heart disease [72,73], structural heart disease [74], CPVT [75], post-shock arrhythmia [64], Brugada and Long QT syndromes, both acquired or drug-induced [3,76,77].

In this study, pro-arrhythmic electrical abnormalities i.e. EADs, DADs and triggered APs were investigated at cellular and fibre scale also including biological variability through the population of models approach. Our simulations report that EADs, DADs and triggered APs are based on different ionic mechanisms, which are consistent with previous investigations in other species [2,14,78,79].

Across all the simulations, EADs were uniquely due to I_CaL_ reactivation, and occurred in models with strong I_CaL_ upregulation, and also with high I_NaL_. This is consistent with simulation results using human ventricular models [31,60]. No I_NaL_ re-activation was observed during EADs, although this may occur using a different protocol [50]. Simulations in tissue confirmed that EADs are able to propagate along the whole fibre and demonstrated the possibility of using the model to investigate PCs as arrhythmia trigger in tissue and whole-organ simulations.

Moreover, across the population of models, DADs generation required Ca^2+^ accumulation both in the SR and in the intracellular space at fast pacing (Fig. 5B). DADs and triggered APs in PCs have been clinically related to arrhythmia initiation [1]. They are suspected to trigger postshock arrhythmias such as ventricular tachycardia and fibrillation after a successful defibrillation of the heart [52]. The mechanisms underlying DADs have been difficult to unravel, but many experimental and simulation studies identified the important role of Ca^2+^ dynamics [1]. In general, experiments across a wide range of protocols, suggested that the key feature for DADs generation is Ca^2+^ overload, though the mechanisms necessary for DADs are still being debated [53]. Our study suggests a similar mechanism also in human Purkinje cells, and identifies I_CaL_ and I_NCX_ as the ionic currents playing a major role in DADs inducibility, and I_K1_ and I_NaK_ for triggered APs. The present study helps in the translation from animal to human studies, and provides a new and needed tool for in silico investigation of arrhythmia, including the Purkinje system and its electrophysiology.

### 4.3 Limitations

The Trovato2020 model presented in this study was built using the human ventricular ORd model, the Purkinje-specific Ca^2+^ sub-system, cellular compartments and intracellular ionic fluxes of the canine PRd model and experimental human data. The model was calibrated using the only currently available dataset from undiseased human PC and validated against an independent experimental dataset from human undiseased hearts in control and with dofetilide, not used for the model calibration and optimisation. The latter also represent the first published dataset from undiseased human PCs under drug action. We cannot exclude the possibility that connexin-mediated electrotonic interactions between PCs and ventricular tissue might have affected the AP waveforms of our experimental datasets, in particular, during the early repolarisation phase. A limitation of this study is the lack of experimental data on the Ca^2+^ transients in human cardiac PCs, since there are no recordings available. Data on human PCs AP response to pure I_CaL_ modulations are also lacking. However, previous studies using animal Purkinje and human ventricular preparations suggest positive correlation between APD_90_ and I_CaL_ modulation. Preparations used in Dataset I were obtained from failing hearts, which could affect I_to_, I_sus_, I_K1_ measurements. This uncertainty was tackled in our study through a population of models investigation using hundreds of models. During the model development, we used the original ORd human ventricular current formulation of currents, whose data were no available for human PCs (e.g., I_Ks_, I_NCX_, I_NaK_). This is further supported by the fact that there are no reports on different isoforms in human ventricular versus Purkinje cardiomyocytes. I_f_ and I_CaT_ formulations were adopted from the canine PRd model. However, I_CaT_ does not play an important role in PCs dynamics and abnormalities, and there are no reports of significant differences between canine and human I_f_ in PCs.

## Author contributions

CT, EP, SS, BR conceived and designed the study; CT and EP designed the models. CT performed the simulations, analysed the data, prepared the figures and drafted the manuscript; NN and AV provided the experimental data for Dataset II; NAG provided the experimental data for Dataset III. CT, EP and BR interpreted the results; all the authors edited and revised the manuscript, and approved the final version.

## Declaration of Competing Interest

CT, EP, NN, AV, SS and BR declare no conflicts of interest. NAG is an employee of AnaBios Corporation.

## Appendix A Supplementary data

Supplementary material Image 1
