# Supplementary material for: Human Purkinje in silico model enables mechanistic investigations into automaticity and pro-arrhythmic abnormalities
Source: J Mol Cell Cardiol. 2020 May;142:24–38. doi: 10.1016/j.yjmcc.2020.04.001 (PMC7294239; doi:10.1016/j.yjmcc.2020.04.001)
Supplement: Supplementary file 1 — Supplementary material [file mmc1.pdf]

Supplementary Material

Title: *"Human Purkinje in silico model enables mechanistic investigations into automaticity and pro-arrhythmic abnormalities"*

C. Trovato, E. Passini, N. Nagy, A. Varró, N. Abi-Gerges, S. Severi and B. Rodriguez.

Index

Page

1. Supplementary Methods

1.1 The Calibrated Trovato2020 Model 4
1.2 Sensitivity Analysis 5
1.3 Optimisation with a Multi-Objective Genetic Algorithm 6
1.4 1D Purkinje Fibre 7

2. Supplementary Tables

Table S1. Parameters and ranges to perform sensitivity analyses and model optimisation trough multi-object genetic algorithm 8
Table S2. Normalised relative correlation coefficients for each ionic current and simulated AP biomarker at 1 Hz 9
Table S3. Comparison experimental and simulated AP biomarkers with different cardiac models at 1Hz 10
Table S4. Effects of simulated I_{Na} blocks on the maximum depolarisation velocity and conduction velocity in cell and fibre 11

3. Supplementary Figures

Figure S1. Overview of Dataset II 12
Figure S2. Sensitivity analysis performed on the initial model - Conductances 13
Figure S3. Sensitivity analysis performed on the initial model - Kinetics 14
Figure S4. Models obtained through optimisation with multi-objective genetic algorithm 15
Figure S5. The optimised Trovato2020 model at 1 Hz 16
Figure S6. Comparison with relative human cardiac models 17
Figure S7. CaMKII effects on rate dependence and DADs 18
Figure S8. DADs mechanisms investigation 19

4. Sensitivity Analysis - Optimised Trovato2020 model

Figure SA1. Single current conductances modulation at 1Hz 21
Figure SA2. Simulation of the APD90 rate-dependence behaviour in response to single current conductances modulation 22
Figure SA3. Simulated AP responses to selective channel blockers at different concentrations 23
Figure SA4. Simulated APs at slow pacing and with 85% IKr block - EADs Protocol 24
Figure SA5. Simulated APs at fast pacing in control and with RyR hypersensitivity - DADs Protocol 25

5. Model Parameters and Equations

Stimulus, Extracellular Concentrations, Cell Geometry, Steady State conditions 26
Maximum Current Conductances, Calcium Buffer Constants 27
Voltage 28
Fast-Sodium Current (I_{Na}) 28
Late-Sodium Current (I_{NaL}) 29
L-type Calcium Current (I_{CaL}) 29
T-type Calcium Current (I_{CaT}) 31
Transient Outward Current (I_{to}) 32
Sustained Potassium Current (I_{sus}) 32
Rapid Delayed Rectifier Potassium Current (I_{Kr}) 32
Slow Delayed Rectifier Potassium Current (I_{Ks}) 33
Hyperpolarization-activated Current (I_f) 33
Inward Rectifier Potassium Current (I_{K1}) 33
Sodium-Calcium Exchange Current (I_{NaCa}) 33
Sodium-Potassium ATPase Current (I_{NaK}) 35
Background currents: I_{Nab}, I_{Cab}, I_{pCa} 36
Calcium/Calmodulin-Dependent Protein Kinase (CaMK) 37
Sarcoplasmic Reticulum Ca^{2+} Fluxes 37
Diffusion Fluxes 39
Ionic Concentrations 39

6. Supplementary References 42

1 - Supplementary Methods

1.1 The Calibrated Trovato2020 Model

Figure 1B illustrates the structure of the new human Purkinje model, the Trovato2020 model, with 6 cellular compartments, 18 trans-membrane ionic currents, 3 intracellular concentrations (Na^+, K^+ and Ca^{2+}) for each of the 3 cellular compartments, Ca^{2+} buffers and CaMKII kinetics as in the ORd model (O'Hara et al. 2011), and Ca^{2+} subsystem as in the PRd model (Li and Rudy 2011). Overall, the Trovato2020 model has 46 state variables.

Cellular compartments: the intracellular compartmentalisation was defined as in the PRd model, based on a triple-layer structure suggested by (Stuyvers et al. 2005). The cell, represented as a cylinder, is divided into 3 cytoplasmic compartments: i) peripheral coupling subspace (SS), where sarcolemmal Ca^{2+} entry (via I_{CaL}) interacts with the sarcoplasmic reticulum (SR); ii) sub-sarcolemmal region (SL), representing the layer of cytoplasm underneath the membrane; iii) bulk myoplasm, that represents the innermost region of the cell. The SR is also divided into 3 compartments:

i) junctional SR, with two Ca^{2+}-release units (RyR3 and IP_3R), which respond to Ca^{2+} changes in the SS;
ii) corbular SR, representing the SR portion close to the cell membrane, and with Ca^{2+}-release units (RyR2) which respond to Ca^{2+} changes into the bulk myoplasm only;
iii) network SR , representing a region between JSR and CSR with no expression of Ca^{2+}-release units (Li and Rudy 2011).

Potassium Currents: I_{to}, I_{sus} and I_{K1} formulations were constructed using Dataset I (Han et al., 2002) as follows. I_{to} formulation includes one activation gate and two inactivation gates (fast and slow) with steady state activation/inactivation curves and inactivation time constants, larger in PCs than in VCs, based on experiments by Han et al., 2002, and activation time constant as in the ORd model (no experimental data available). Thus, the Trovato2020 representation of I_{to} differs both from the ventricular formulation used by ten Tusscher and Panfilov 2008 and from the one implemented in Stewart et al. 2009. In particular, the latter describes I_{to} inactivation using only one gate, using experimental data for the slow inactivation time constant published by (Han et al. 2002), whereas, the activation time constant is fitted on the experimental data for I_{to} fast inactivation. The small ORd background K^+ current was replaced with a single instantaneous voltage-dependent current, based on the experimental I-V curve for I_{sus}. The I_{K1} formulation was modified by fitting the instantaneous voltage-dependent

rectification gate to the experimental I-V curve, shifted by -14 mV to account for the liquid junction potential and to match the reversal potential to the potassium Nerst potential computed from the intra and extra-cellular concentrations. The maximum conductances of these three currents were also set based on the experimental I-V curves, after correcting the data for heart failure (+25% and +56% for I_{K1} and I_{to}, respectively), as described in (Stewart et al. 2009). I_{Kr} and I_{Ks} formulations were left as in the ORd model, since no human Purkinje data are available in literature (Nagy et al. 2015) and gene expression levels for these proteins in humans (hERG and KvLQT1) were found to be similar in PCs and VCs (Gaborit et al. 2007). Based on the sensitivity analysis, some changes were made to I_{Kr} kinetics: activation time constants were shifted by +15 mV and the time-independent inactivation gate was scaled by a factor of 0.3 (Table S1).

Sodium Currents: The Trovato2020 model includes the modified ORd formulation of I_{Na} described in (Dutta et al. 2017; Passini et al. 2016) which supports AP propagation also in 3D simulations. Several studies in PCs (Haissaguerre et al. 2016; Nagy et al. 2015) highlighted the role of non-cardiac isoforms of the sodium channel, which have slower inactivation kinetics and a different sensitivity to tetrodotoxin. In order to account for the sodium current characteristics and functional role experimentally shown in PCs, the original ORd formulation of I_{NaL} was kept in the Trovato2020, though its conductance was increased 2.5 folds, based on experimental evidence by (Iyer et al. 2015; Haufe et al. 2005). However, we did not split the formulation into cardiac and non-cardiac isoform contributions, which might be a possible future development when data from human PCs become available. Background Na^{+} current was increased by the same amount as I_{NaL}, as in (Passini et al. 2016).

Calcium Currents: I_{CaL} formulation was left as in the ORd model, since no human Purkinje data are available in literature (Nagy et al. 2015). Based on the sensitivity analysis, some changes were made to the current kinetics: both inactivation and activation gates were shifted by +2 mV, and inactivation time constants were scaled of 30% and shifted by +15 mV (Table S1). I_{CaT} was incorporated into the model, using the formulation proposed in the PRd model. Background Ca^{2+} current (I_{Cab}) and the sarcolemmal Ca^{2+} pump (I_{pCa}) were left as in the ORd model.

Other currents: I_f was included in the model, using the formulation proposed in the PRd model. I_{NCX} and I_{NaK} were kept as in the ORd model.

1.2 Sensitivity Analysis

A sensitivity analysis was conducted on the initial model to investigate how the 6 criteria for

model calibration are affected by variations in the conductances of all the ionic currents (I_{Na}, I_{NaL}, I_{CaL}, I_{CaT}, I_{to}, I_{sus}, I_{Kr}, I_{Ks}, I_f, I_{K1}, I_{NCX}, I_{NaK}) and in the kinetics of I_{CaL} and I_{Kr} (I_{CaL}: steady state activation and inactivation, fast and slow inactivation time constants; I_{Kr}: fast and slow activation time constants, steady state inactivation). Each current conductance was varied from 10% to 200% of its nominal values, while specific variation ranges were defined for each current kinetics parameter (Table S1). For each value of each parameter, the protocols 1-6 were simulated to evaluate the effects of the current modulation on each of the 6 criteria used for the model design. A sensitivity analysis was also performed for the optimised model (see below, Section 1.3), varying only the current conductances. Correlation coefficients between ionic currents and AP biomarkers were computed at 1 Hz, similarly to (Romero et al. 2009). For each biomarker b_i, current conductance g_j and conductance scaling factor x the changes in respect to control ($\Delta B_{i,j,x}$) and the sensitivity coefficient ($S_{i,j}$) were computed as follows:

$$\Delta \mathrm{B}_{i,j,x} = b_{i,j,x} - b_{i,j,1} \qquad \begin{cases} 1 \leq i \leq 9 & \textit{AP biomarkers} \\ 1 \leq j \leq 12 & \textit{conductances} \\ 0.1 \leq x \leq 2 & \textit{scaling factors} \end{cases}$$

$$\Delta \mathrm{B}_{i,MAX} = \max_{j,x} \Delta \mathrm{B}_{i,j,x}; \qquad \Delta \mathrm{B}_{i,MIN} = \min_{j,x} \Delta \mathrm{B}_{i,j,x};$$

$$\mathrm{S}_{i,j} = \frac{\Delta \mathrm{B}_{i,j,2} - \Delta \mathrm{B}_{i,j,0.1}}{\max\{|\Delta \mathrm{B}_{i,MAX}|; |\Delta \mathrm{B}_{i,MIN}|\}}$$

where $b_{i,j,x}$ is the value of biomarker b_i when g_j = x; $b_{i,j,1}$ is the value of biomarker b_i using the baseline model; $\Delta \mathrm{B}_{i,MAX}$ and $\Delta \mathrm{B}_{i,Min}$are the maximum and the minimum changes for each biomarker b_i, respectively.

1.3 Optimisation with a Multi-Objective Genetic Algorithm

A multi-objective genetic algorithm (Matlab function *gamultiobj*) was used for automated multi-object optimisation (Deb 2001) of the calibrated model described above. All the ionic current conductances (except for the background currents) were allowed to vary in the range [50-150]% of their nominal values, to exclude extreme up/down-regulations. The 7 kinetic parameters of I_{CaL} and I_{Kr} were allowed to vary in the ranges reported in Table S1, based on the sensitivity analysis results. The algorithm was run for 30 generations, with 300 models each. The multi-object cost function was computed as a weighted sum of 2 error functions: i) distance from the experimental mean of the AP biomarkers in control condition at 1 Hz (Dataset II, Section 2.1 in the main text); ii) a linear combination of errors, based on the criteria 2-5 described in the main text (Section 2.2) for the APD_{90} rate-dependence, AP response to I_{CaL} and I_{Kr} modulations and EADs inducibility. At the end of each generation, the Pareto front

(Deb 2001) was computed, to identify the 35% of local minima of the cost function, i.e., the models with the best performances, then combined to create the following generation. After 30 generations, the model with best performance was chosen as the final optimised Trovato2020 model.

1.4 1D Purkinje Fibre

In order to evaluate the effects of intracellular coupling on the protocols tested (Section 2.5 in the main text), a 1D Purkinje fibre model was constructed using the Trovato2020 model to represent membrane kinetics. The 5 cm fibre was discretised in 100 nodes to obtain a spatial resolution of 500 µm. Temporal integration step was set to 500 µs. Both spatial and temporal resolutions where determined to guarantee the best compromise between spatial and temporal convergence errors (<10%) (Bueno-Orovio et al. 2014) and computational time for simulations. The monodomain formulation was used to simulate propagation along the fibre (Keener and Sneyd 2009) and was solved using the Fourier spectral method for fractional diffusion (Bueno-Orovio et al. 2014). The Rush-Larsen method (Rush and Larsen 1978) was implemented for the integration of the gating variables to speed up the simulations. Stimulus duration was set to 2.2 ms, i.e. twice the minimum value to achieve propagation in the fibre. The fibre was stimulated on one side for 1 mm. For each protocol tested, three beats were simulated in the fibre, to allow relaxation from the initial conditions (all the nodes were initialised to the SS computed at cellular level). APD_{90} and CV were computed for the last beat. CV was computed at the centre of the fibre, to avoid border effects, as the distance between the 17 central nodes (set to 0.26 cm), divided by the difference of activation times (identified as the instant with maximum dV/dt) at the border nodes.

Diffusion coefficient was set to 9 cm^2/s to match the CV of 1.6 m/s computed from observations at sinus rhythm in human Purkinje fibres (Kupersmith et al. 1973, Durrer et al. 1970). Protocols 1, 5, 6 and 8 (Section 2.5) were simulated in fibre. In addition, effects of I_{Na} blocks on the CV were considered, modifying the protocol used for hERG-blocks. I_{Na} was reduced up to 100%, or until propagation failure was observed.

2 - Supplementary Tables

Table S1. Parameters and ranges to perform the sensitivity analysis and to optimise the calibrated model trough multi-object genetic algorithm

	Sensitivity analysis range		Calibrated model Pre-optimisation Coefficients	Optimisation Range		Final Model Post-optimisation Coefficients
	Min	Max		Min	Max	
			Conductances			
**G_{Na}**	[10 ; 200] %		1	[50 ; 150] %		**0.75**
**G_{NaL}**			2.5*			**1**
**G_{CaL}**			1			**0.75**
**G_{CaT}**			1			**0.96**
**G_{to}**			1			**0.93**
**G_{sus}**			1			**1.28**
**G_{Kr}**			1			**0.93**
**G_{Ks}**			1			**0.84**
**G_{f}**			1			**0.97**
**G_{K1}**			1			**0.67**
**G_{NCX}**			1			**1.2**
**G_{NaK}**			1			**1.1**
			I_{CaL} Kinetics			
**Activation/ Inactivation V shift (mv)**	-4	4	2	0	4	**3.3**
**Time constants V shift (mv)**	-10	30	15	10	20	**15.2**
**Slow time constants scales**	0.1	3	0.7	0.4	1	**0.49**
**Fast time constants scales**	0.1	3	0.7	0.4	1	**0.72**
			I_{Kr} Kinetics			
**Fast time constant V shift (mV)**	[-10 ; 30] mV		15	10	20	**17.6**
**Slow time constant V shift (mV)**	[-10 ; 30] mV		15	10	20	**17.2**
**Inactivation slope**	[0.3 ; 3]]		0.3	0.1	0.5	**0.32**

Varied parameters (1st column). Ranges investigated via sensitivity analysis (2nd & 3rd columns). Conductances scaling factors and kinetics coefficients for the calibrated model (4th column). Optimisation ranges for the multi-object genetic algorithm (5th & 6th columns). Conductances scaling factors and kinetics coefficients for the final model are reported in bold (7th column). *from (Iyer et al., 2015).

Table S2. Normalised relative correlation coefficients for each ionic current and simulated AP biomarker at 1 Hz.

	I_{Na}	I_{NaL}	I_{CAL}	I_{CaT}	I_{to}	I_{sus}	I_{Kr}	I_{Ks}	I_f	I_{K1}	I_{NCX}	I_{NaK}
$\frac{dV}{dt}$(V/s)	**1.0**								-0.1	0.1		0.2
APD_{90}		0.4				**-0.6**	**-1.0**			-0.1	0.3	
APD_{75}		0.4				**-0.6**	**-1.0**				0.3	
APD_{50}	-0.1	0.5	0.1			**-0.8**	**-1.0**				0.4	
APD_{25}	-0.2	0.4	0.3			**-1.0**	-0.3				0.4	0.1
APD_{10}	-0.5	0.3	0.4		-0.2	**-1.0**						
APA	**1.0**	0.1	0.4		-0.4	**-0.9**	-0.2		-0.3	0.3	0.1	0.4
TOP		-0.1	-0.2			0.2	0.5		**1.0**	**-1.0**	-0.5	**-0.8**
EOP		-0.1	-0.2			0.3	0.4		**1.0**	**-1.0**	-0.5	**-0.8**

Values bigger than 0.60 are highlighted in bold. Values smaller than 0.1 are not shown. The upstroke velocity is set by the I_{Na} and, to a lesser extent, by I_f, I_{K1},and I_{NaK}. The APD at late stages of repolarisation, 50-75-90%, is determined by the contribution of different currents: I_{NaL} and the I_{NCX} prolong the AP; I_{sus} and I_{Kr} shorten it. At the early phase of repolarisation, APD at 10-25%, the effects of I_{NaL} and I_{Kr} are reduced, whereas, I_{Na} and I_{CaL} play a more relevant role in setting the APD. Both I_{Na} and I_{CaL} increase the AP amplitude whereas I_{to} and I_{sus} reduce it. I_{NaK} and, to a lesser extent, I_{K1} and I_f affect the APA indirectly, since they affect the membrane resting potential. The major currents responsible for the membrane repolarisation are, I_{K1} and $I_{NaK,}$ whereas I_f depolarises the cell during the diastolic interval. No relevant changes were induced by the modulation of I_{Ks} and I_{CaT}.

Table S3. Comparison between experimental and simulated AP biomarkers with different cardiac models at 1Hz

BIOMARKER	EXPERIMENTS	TROVATO 2020	STW	TT08	ORD	PRD
$\frac{dV}{dt}$(V/s)	387 ± 143	381	522	742	264	527
APD_{90} (ms)	294 ± 76	306	285	374	268	527
APD_{75} (ms)	261 ± 67	280	254	364	248	328
APD_{50} (ms)	210 ± 52	224	197	330	208	208
APD_{25} (ms)	117 ± 46	143	13	209	167	31
APD_{10} (ms)	33 ± 35	34	4	3	74	2
APA (mV)	106 ± 7	110	120	146	128	134
TOP (mV)	-85 ± 2.4	-86.5	-74.0	-86.0	-87.8	-84.1
EOP (mV)	-86 ± 2	-87.3	-77.3	-86.0	-88.0	-84.9

dV/dt_{MAX}: maximum depolarisation rate; **APD_X:** AP Duration at X% of repolarisation; **APA:** action potential amplitude; **TOP**: take-off potential, voltage level before depolarisation; **EOP:** end of potential, voltage level at the end of repolarisation. **STW**: Stewarts et al. 2009 model; **TT08**: ten Tusscher et al 2008 model; **ORd:** O'Hara et al 2011 model; **PRd:** Pan Li & Rudy 2011 model.

Table S4. Effects of simulated I_{Na} block on the maximum depolarisation velocity and conduction velocity in Purkinje cell and fibre using the Trovato2020 model.

	SINGLE CELL		FIBRE	
**I_{Na} block**	$\frac{dV}{dt}_{Max}$ **(V/s)**	**AP Inducibility**	**CV (cm/s)**	**AP propagation**
**Control (No Block)**	380	Yes, Na^{+} driven	160	Yes
**30%**	315	Yes, Na^{+} driven	147	Yes
**50%**	260	Yes, Na^{+} driven	136	Yes
**90%**	92	Yes, Na^{+} driven	88	Yes
**95%**	44	Yes, Na^{+} driven	69	Yes
**100%**	40	Yes, Ca^{2+} driven	-	No

$\frac{dV}{dt}_{Max}$: maximum depolarisation rate computed in single cell; CV: conduction velocity computed in a 5 cm 1D fibre.

3 - Supplementary Figures

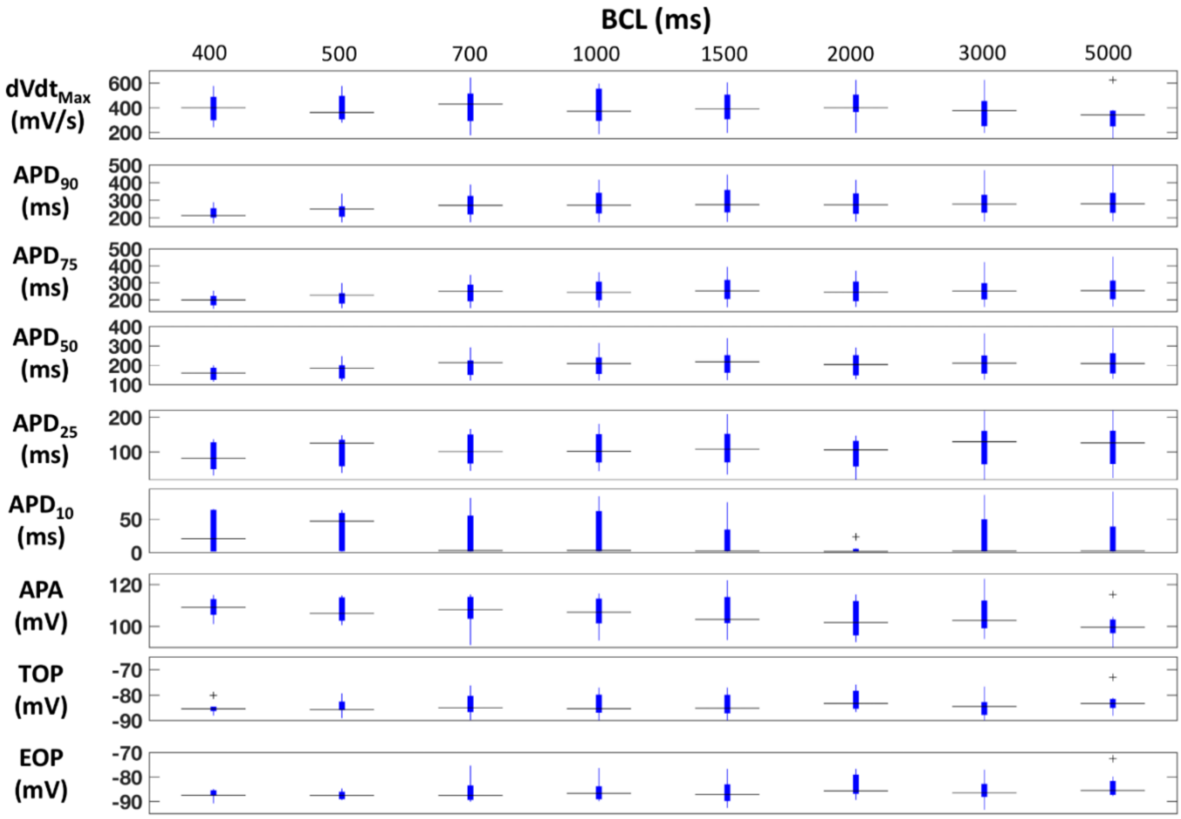

Figure S1. Overview of Dataset II. BCL: basic cycle length. **dV/dtMAX:** maximum depolarisation rate; **APDx:** AP Duration at X% of repolarisation; **APA:** action potential amplitude; **TOP**: take-off potential, voltage level before depolarisation; **EOP:** end of potential, voltage level at the end of repolarisation.

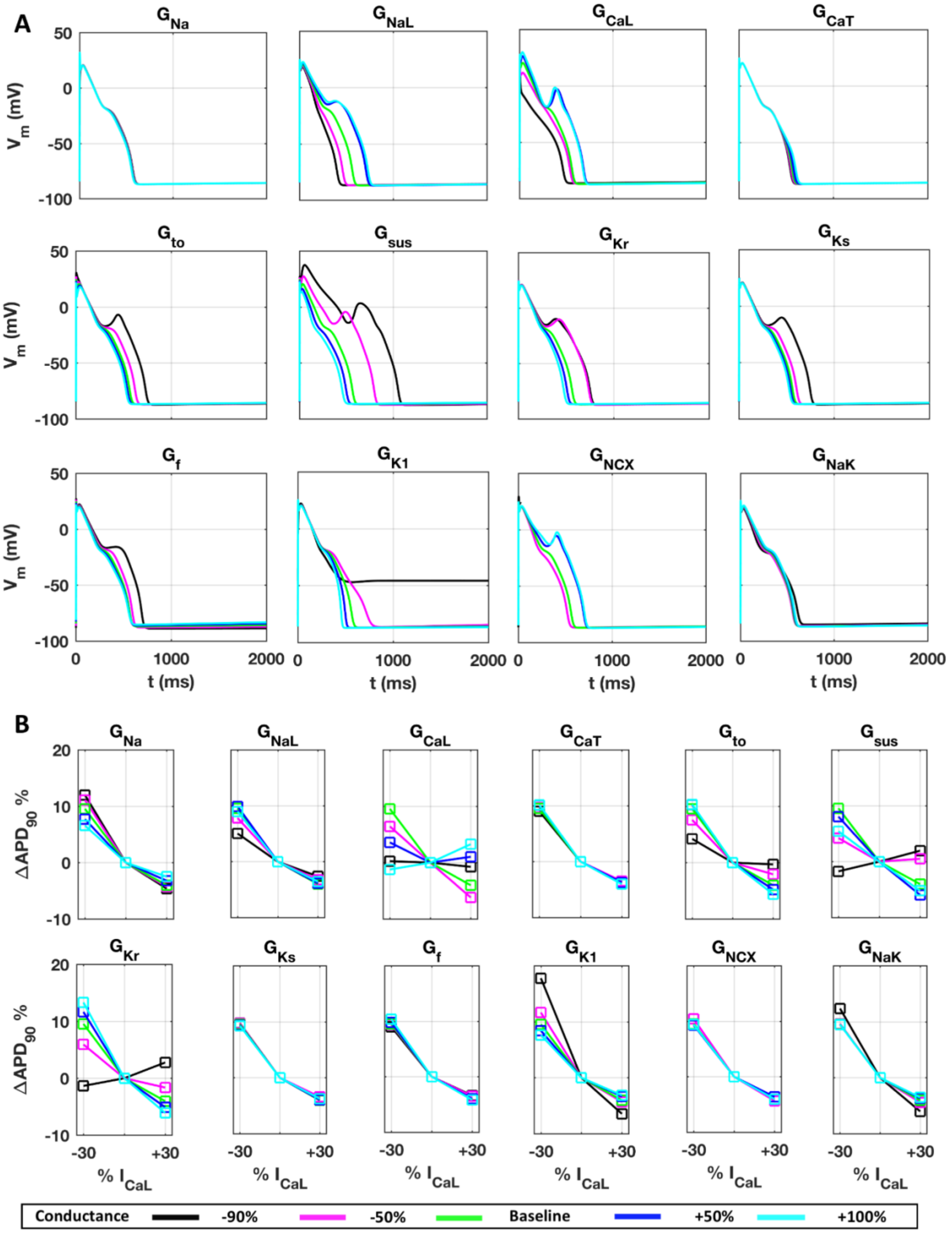

Figure S2. Sensitivity analysis performed on the initial model varying current conductances as reported in Table S1. Investigation of each current contribution in EADs inducibility and APD_{90} response to I_{CaL} modulations. The legend indicates the percentage of modulation for each conductance. A) Simulated APs following Protocol 5 for EADs inducibility. B) Percental changes in the APD_{90} following Protocol 3 in response to I_{CaL} conductance changes (±30%).

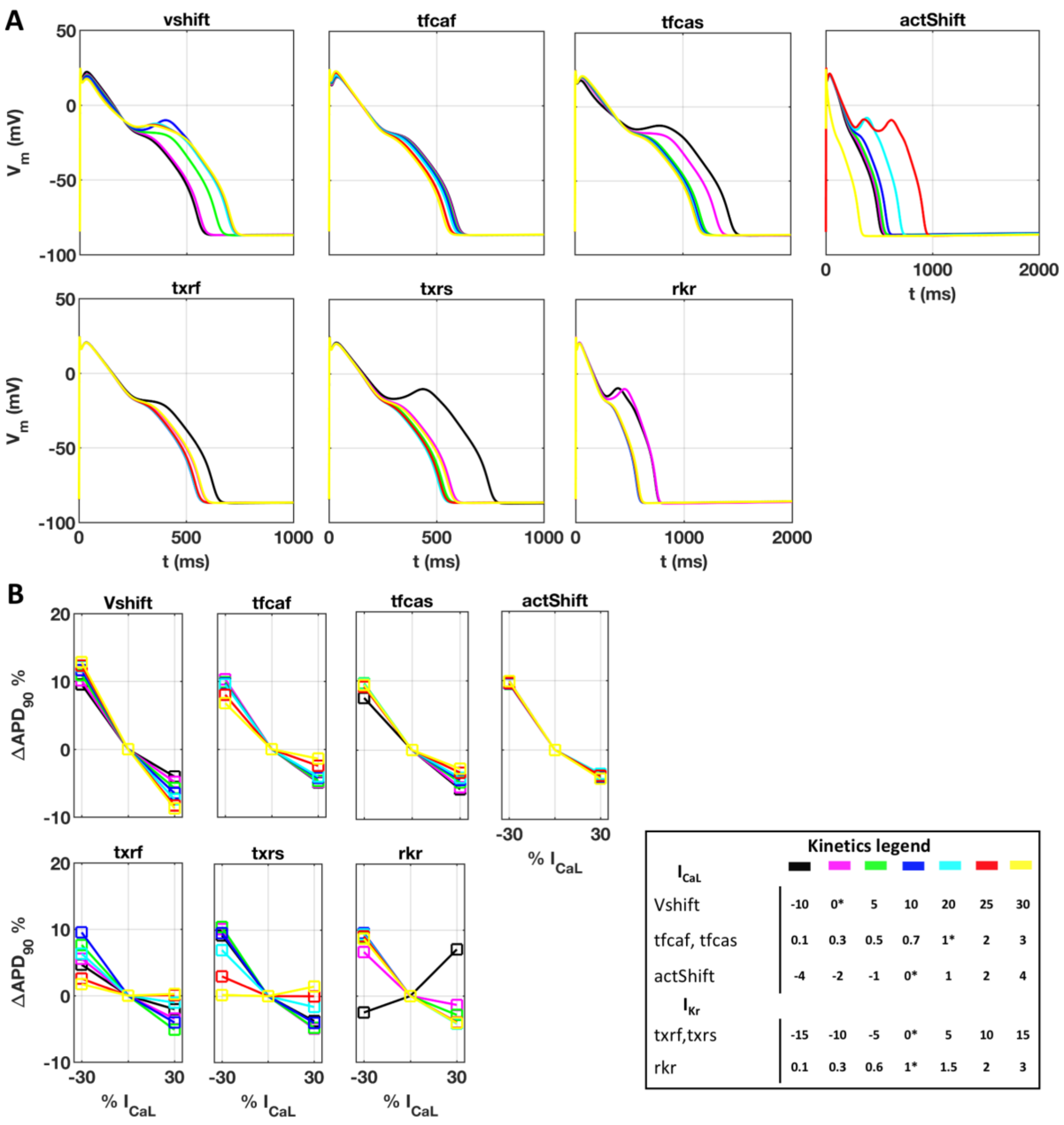

Figure S3. Sensitivity analysis performed on the initial model, varying I_{Kr} and I_{CaL} kinetics, as reported in the legend and in Table S1. Investigation of each current contribution in EADs inducibility and APD_{90} response to I_{CaL} modulations. A) Simulated APs following Protocol 5 for EADs inducibility. B) Percental changes in the APD_{90} following Protocol 3 in response to I_{CaL} changes (±30%). *Nominal value for the initial model.

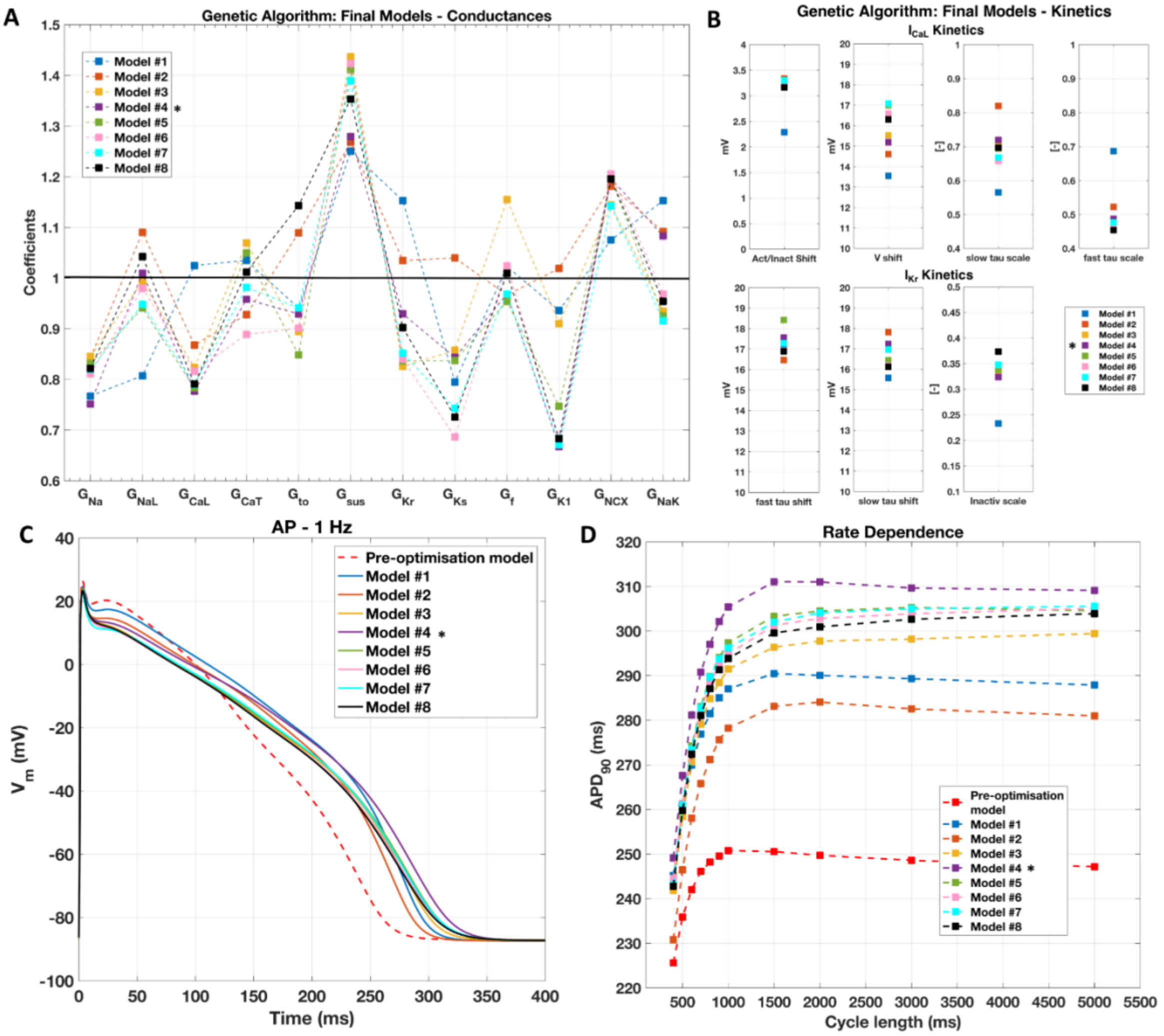

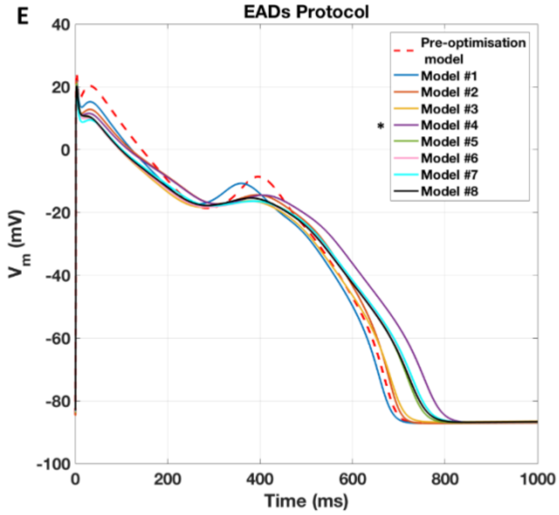

F

Model Nº	ΔAPD_{90}		
	$-30\%\ I_{Kr}$	$-30\%\ I_{CaL}$	$+30\%\ I_{CaL}$
Pre-Opt	+20%	-2.9%	+3.1%
#1	+15%	-1.9%	+2.2%
#2	+19%	-0.6	+1.3%
#3	+18%	0	+0.8%
#4	+19%	-0.6%	+0.9%
#5	+19%	-0.3%	+0.7%
#6	+19%	+0.5%	+0.6%
#7	+19%	+0.6%	+0.5%
#8	+20%	+0.5%	+0.6%

Figure S4. Models obtained through optimisation with the multi-objective genetic algorithm. Parameter set for the 8 final models for: A) current conductances B) I_{CaL} and I_{Kr} kinetics. Ranges are definite as in Table S1. C) Simulated APs at 1 Hz. D) APD_{90} rate dependence. E) Simulated APs to induce EADs. F) APD_{90} changes induced by I_{Kr} block or I_{CaL} modulation. *Final Trovato2020 model.

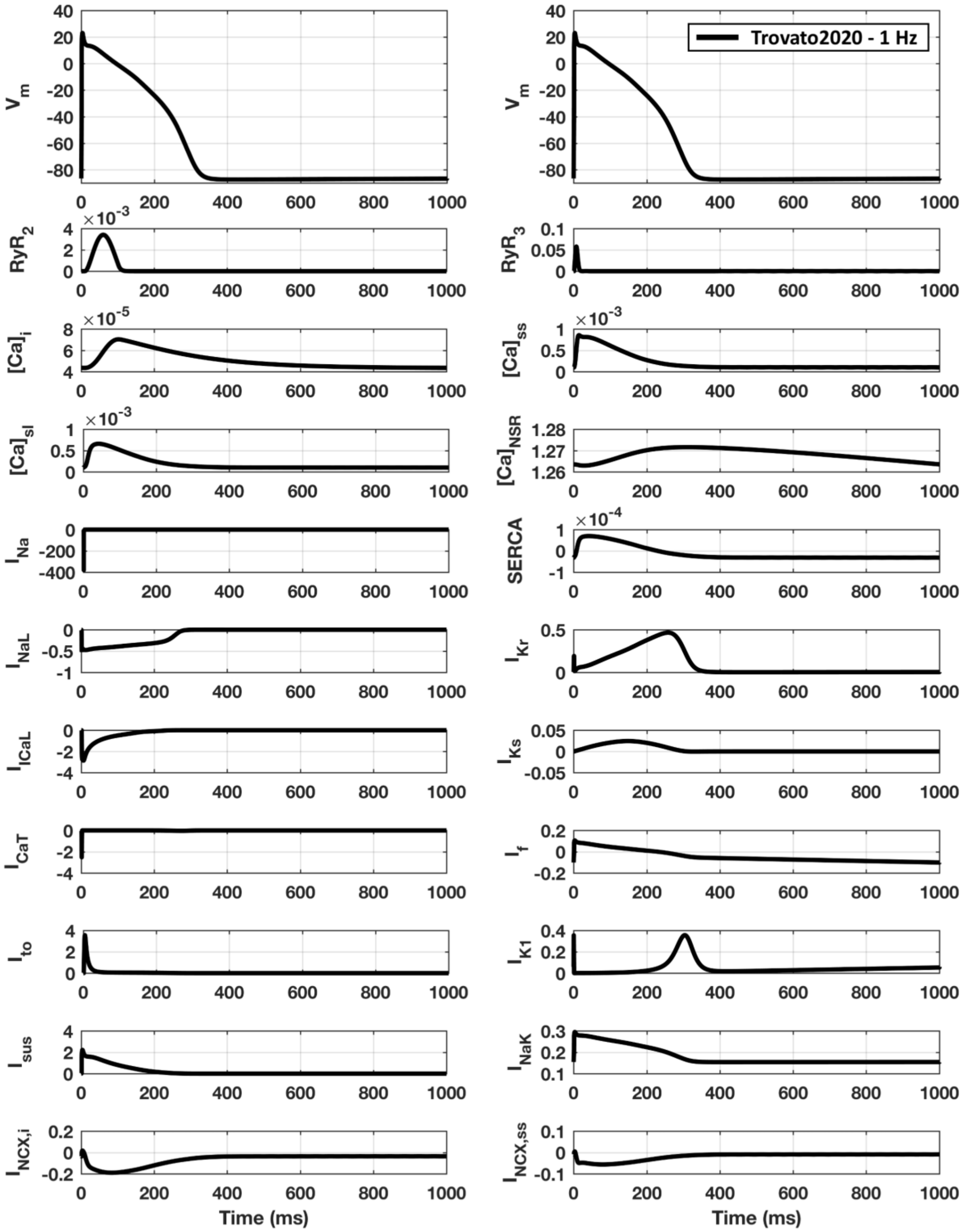

Figure S5. The new Trovato2020 model: simulated AP at 1 Hz in control condition, with the underlying ionic currents, pumps, exchangers, intracellular Ca^{2+} concentrations and fluxes.

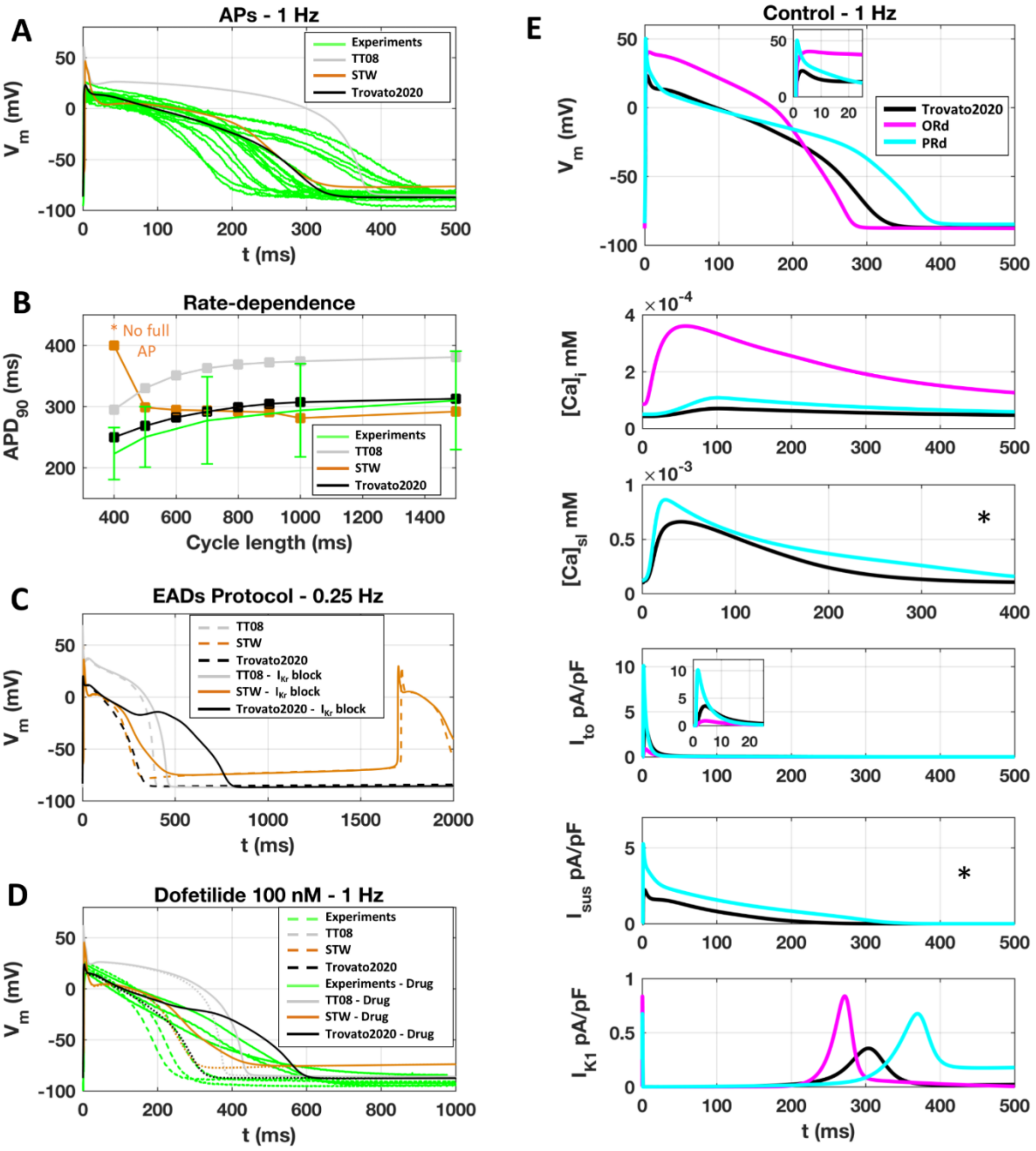

Figure S6. Comparison of different cardiomyocytes models. A) Human Purkinje: TT08 in grey (ten Tusscher and Panfilov 2008), STW in orange (Stewart et al. 2009), the new Trovato2020 model (black) and experiments (green). APs comparison at 1 Hz in control; B) APD90 rate-dependence; C) EADs protocol: 85% I_{Kr} block, pacing at BCL=4000 ms. Control (dash line), AP with I_{Kr} block (solid line); D) AP response to Dofetilide 100 nM: control (dash line), AP with Dofetilide (solid line). E) APs, intracellular and submembrane [Ca^{2+}], and refitted K^+ currents, I_{to}, I_{K1}, I_{sus} for Trovato2020 (black), ORd (O'Hara et al. 2011, pink) and PRd (Li and Rudy 2011, light blue) models. *Not implemented into ORd.

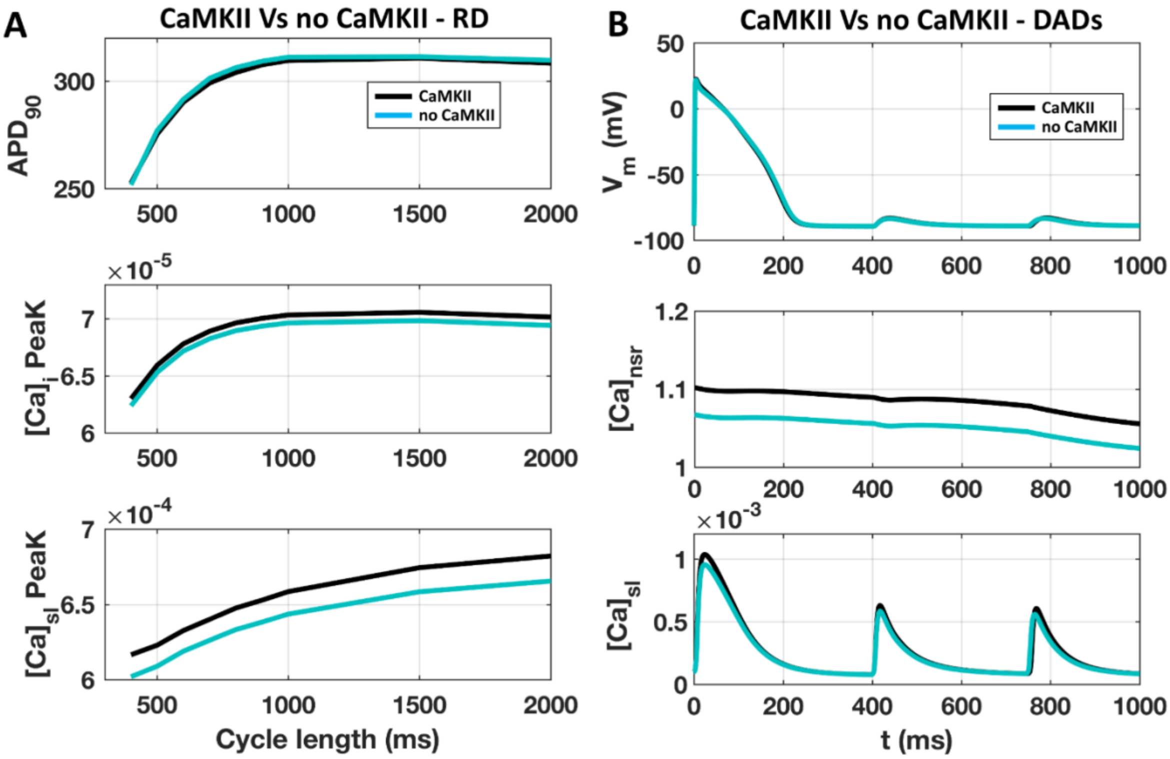

Figure S7. Effects of CaMKII signalling on the Trovato2020 model: control (black) and no CaMKII signalling (light blue). A) APD_{90} rate-dependence, $[Ca^{2+}]_i$ and $[Ca^{2+}]_{sl}$ peaks. B) DADs protocol, fast pacing with RyR hypersensitivity.

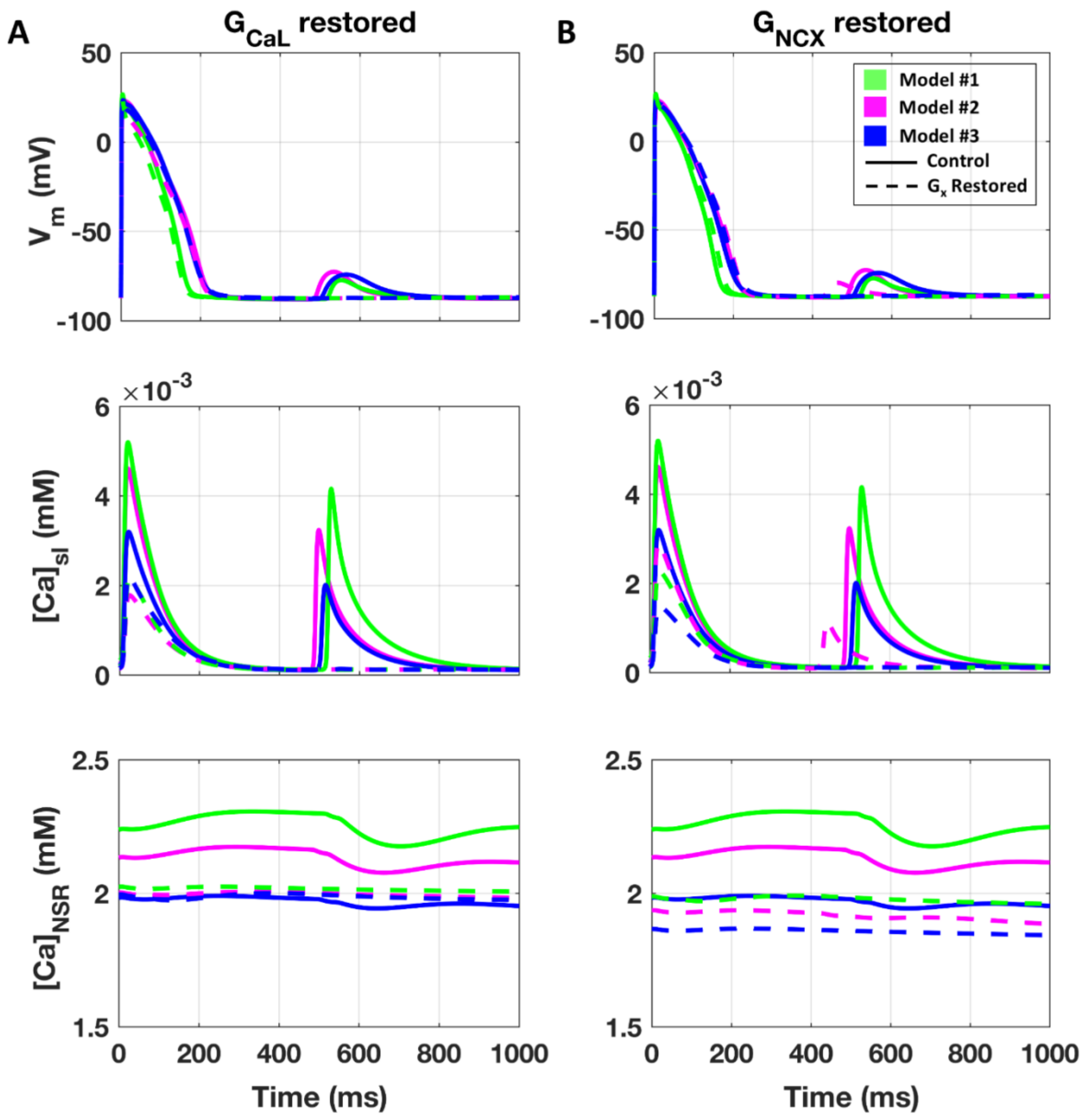

Figure S8. Simulated APs, $[Ca^{2+}]_{SL}$ and $[Ca^{2+}]_{NSR}$ for a selection of 3 models (green, pink and blue traces) from the population producing DADs, in control (solid line) and with selective conductance restored to the corresponding baseline value (dashed line): A) G_{CaL}; B) G_{NCX};

Optimised model
Trovato2020

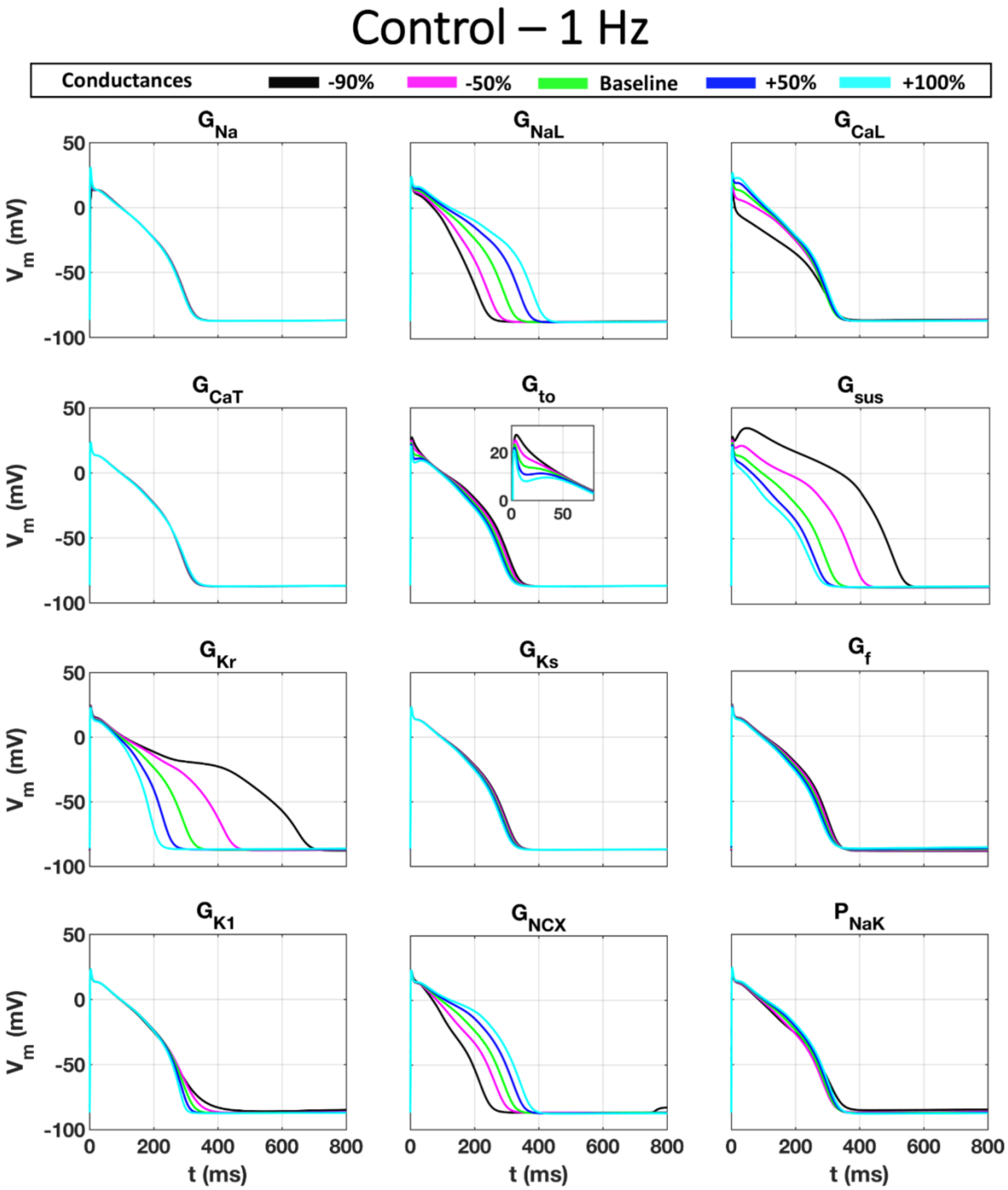

Figure SA1. Single current conductances modulation: simulated APs of the optimised new Trovato2020 model, following Protocol 1 for each changed conductance in the range [0.1 - 2]% of their nominal value (Table S1).

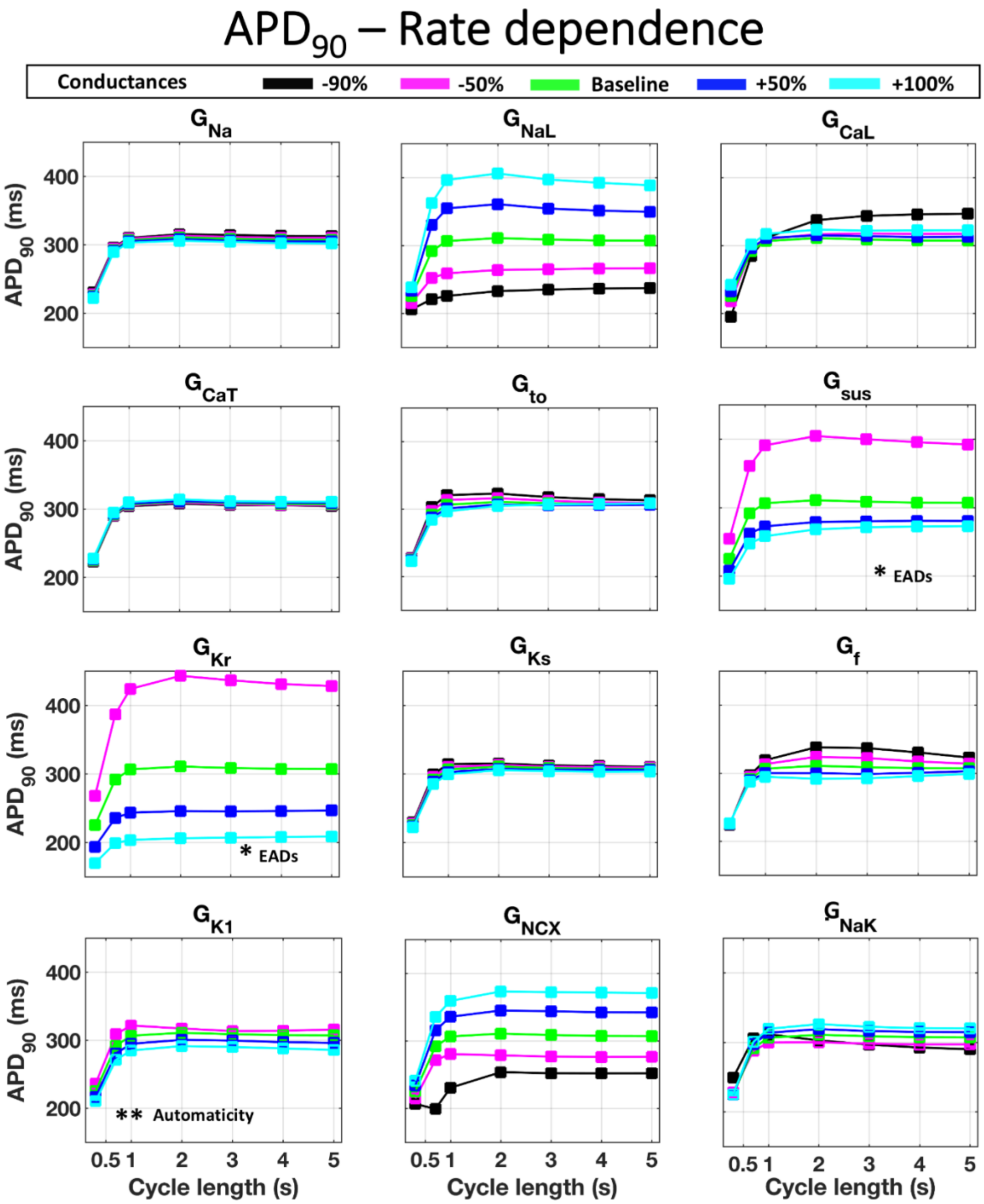

Figure SA2. Simulation of the APD90 rate-dependence behaviour in response to single current conductances modulation in the range [0.1 - 2]% of their nominal value (Table S1) using the optimised new Trovato2020 model. *EAD were induced in case of slow pacing rate with I_{sus} and I_{Kr} downregulation (-90%). **Automaticity was observed at slow pacing rate with IK1 downregulation (-90%).

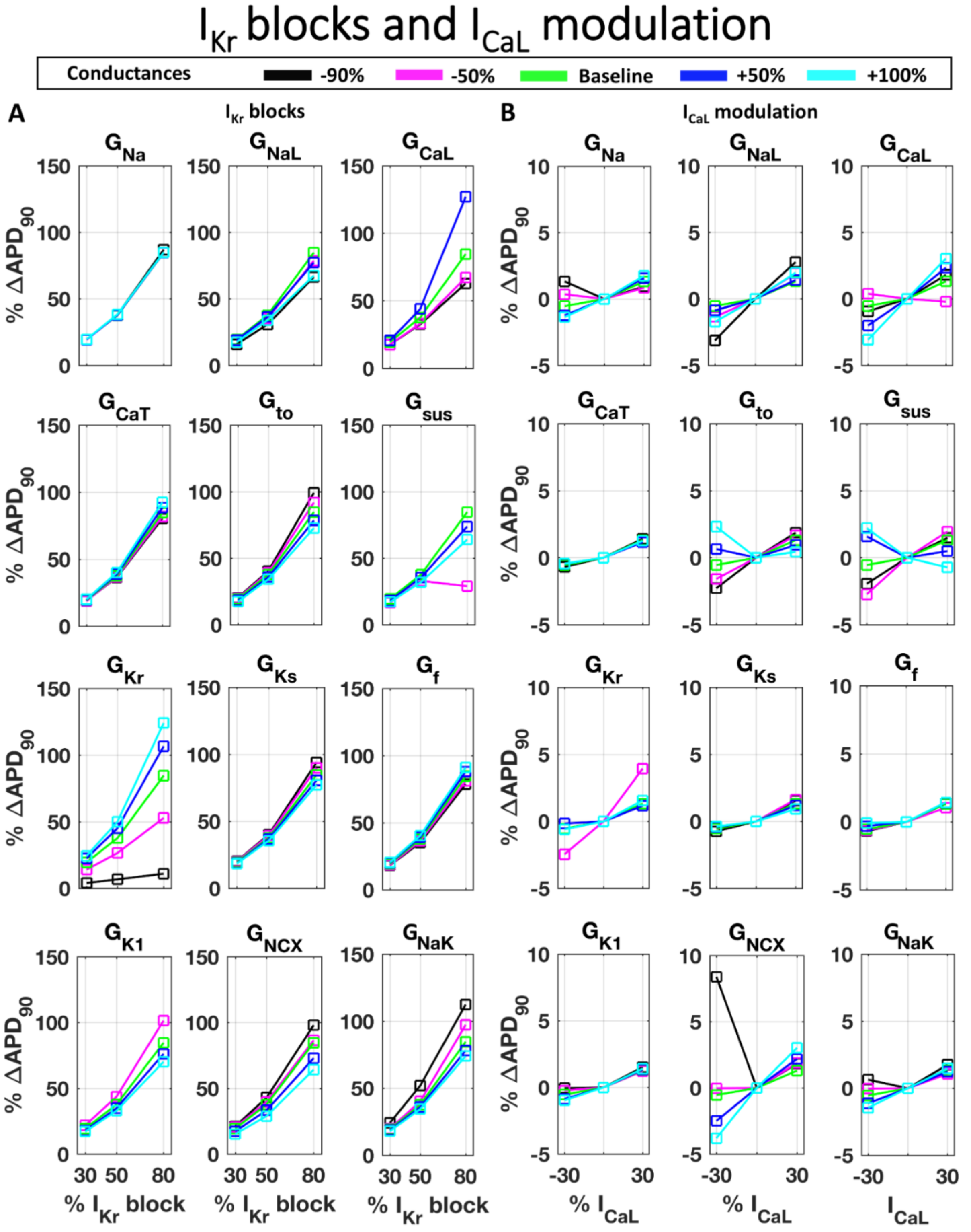

Figure SA3. Simulated AP responses to selective channel blockers at different concentrations and conductances modulation in the range [0.1 - 2]% of their nominal value (Table S1) using the optimised Trovato2020 model: A) I_{Kr} block at 30%, 50% and 80%; B) ± 30% I_{CaL} modulation.

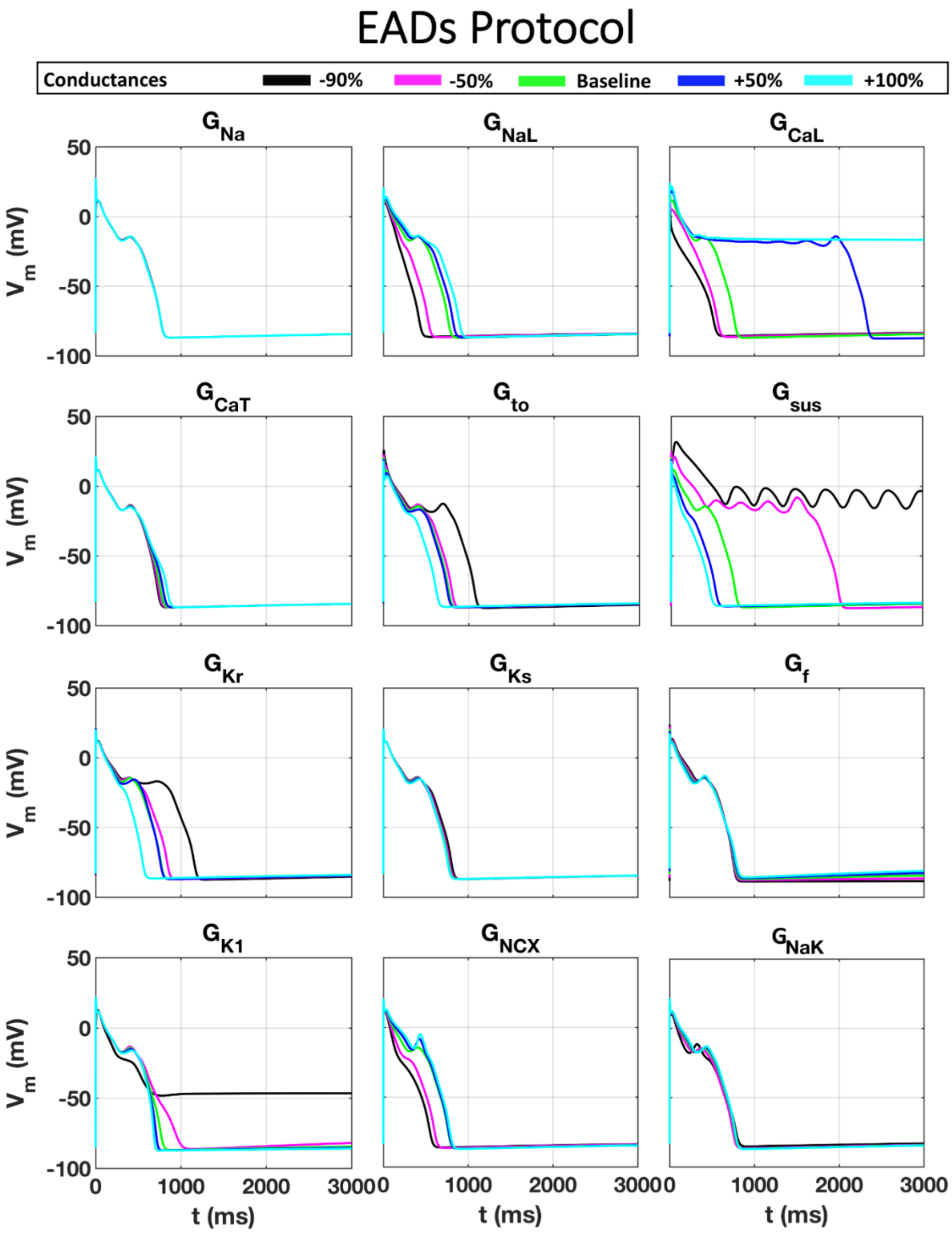

Figure SA4. Simulated APs at slow pacing and with 85% I_{Kr} block and selective conductances modulation in the range [0.1 - 2]% of their nominal value (Table S1) using the optimised new Trovato2020 model. EADs are enhanced by increased I_{CaL}, I_{NaL}, or I_{NCX} and reduced I_{to}, I_{sus}, or I_{Kr}, while modulation of the other currents (I_{Na}, I_{CaT}, I_{Ks}, I_f, I_{K1} and I_{NaK}) do not seem to play a significant role.

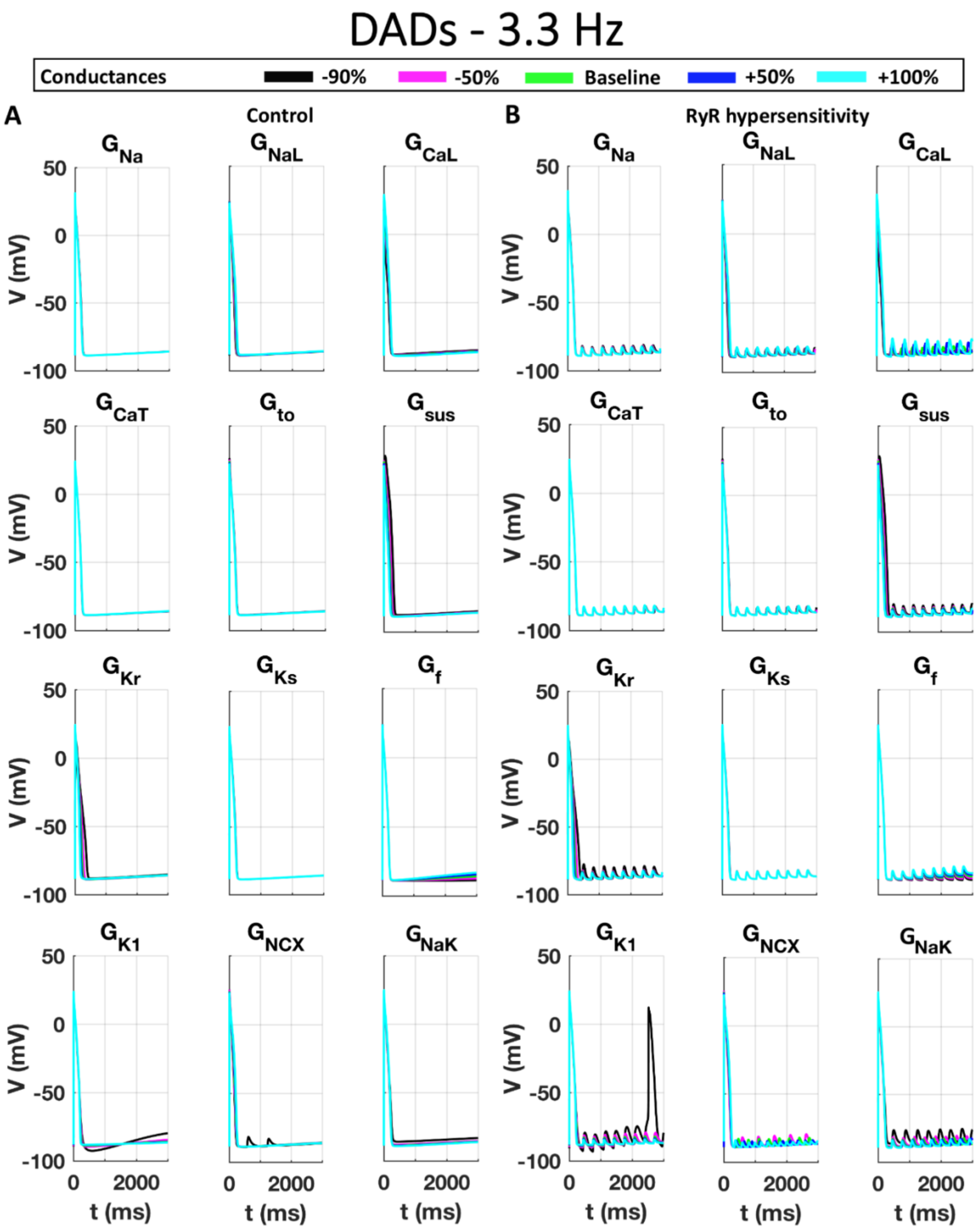

Figure SA5. Simulated APs at fast pacing in control (A) and with RyR hypersensitivity (B) and selective conductances modulation in the range [0.1 - 2]% of their nominal value (Table S1) using the optimised new Trovato2020 model. In control, DADs were observed only with high I_{NCX} downregulation and in every case when including RyR hypersensitivity.

5 - Model Parameters and Equations

Stimulus

Amplitude $I_{\text{stim}} = -40\frac{\mu A}{\mu F}$ Duration= 1ms.

Extracellular Concentrations

$[Na^+]_o = 140\ mM;$ $[Ca^{2+}]_o = 1.8\ mM;$ $[K^+]_o = 5.4\ mM$

Cell Geometry

$L = 0.0164\ cm$ $r = 0.00175\ cm$

$A_{\text{geo}} = 2\pi \cdot r^2 + 2\pi \cdot r \cdot L = 2 \cdot 10^{-4}\ cm^2$ $A_{\text{cap}} = 2 \cdot A_{\text{geo}} = 4 \cdot 10^{-4}\ \mu L$

$v_{\text{cell}} = \pi \cdot r^2 \cdot L = 16 \cdot 10^{-5}\ \mu L$

$v_{\text{myo}} = 0.6 \cdot vcell = 9.5 \cdot 10^{-5}\ \mu L$ $v_{\text{nsr}} = 0.04 \cdot vcell = 6.3 \cdot 10^{-6}\ \mu L$

$v_{\text{ss}} = 0.02 \cdot vcell = 3.1 \cdot 10^{-6}\ \mu L$ $v_{\text{jsr}} = 0.002 \cdot vcell = 3.1 \cdot 10^{-7}\ \mu L$

$v_{\text{sl}} = 0.15 \cdot vcell = 2.4 \cdot 10^{-5}\ \mu L$ $v_{\text{csr}} = 0.008 \cdot vcell = 1.3 \cdot 10^{-6}\ \mu L$

Steady State conditions at 1Hz (after 1000 beats)

1	V =86.55;	j = 0.8;	ffp = 1;
2	$[Na^+]_I$ =8.23;	hsp = 0.6 ;	fcafp = 1;
3	$[Na^+]_{SL}$ =8.23;	jp = 0.8;	b = 0;
4	$[Na^+]_{SS}$ =8.23;	mL = 0;	g = 1;
5	$[K^+]_I$ =144;	hL = 0.5;	xrf = 0;
6	$[K^+]_{SL}$ =144;	hLp = 0.2;	xrs = 0.6;
7	$[K^+]_{SS}$ =144;	a = 0;	xs1 = 0.2;
8	$[Ca^{2+}]_I$ =4.36e-05;	i = 0.6;	xs2 = 0;
9	$[Ca^{2+}]_{SL}$=1.0e-04;	i2 = 1;	xk1 = 1;
10	$[Ca^{2+}]_{SS}$=1e-04;	d = 0;	y = 0.2;
11	$[Ca^{2+}]_{JSR}$ =1.25;	ff = 1;	CaMKt = 0;
12	$[Ca^{2+}]_{NSR}$ =1.27;	fs = 1;	u = 0.5;
13	$[Ca^{2+}]_{CSR}$ =1.27;	fcaf = 1;	$Jrel_1$ = 0;
14	m = 0;	fcas = 1;	$Jrel_2$ = 0;
15	hf = 0.8;	jca = 1;	
16	hs = 0.8 ;	nca = 0;	

Maximum Current Conductances (mS/µF)

G_{Na}	39.46
G_{NaL}	0.0189
G_{CaL}	7.7677e-05
G_{CaT}	0.0754
G_{to}	0.192
G_{sus}	0.0301
G_{Kr}	0.0342
G_{Ks}	0.0029
G_{fNa}	0.0116
G_{fK}	0.0232
G_{K1}	0.0455
G_{NaK}	32.4872
G_{NCX}	9.5709e-04

Calcium Buffer Constants

BSR_{MAX}	0.019975
Km_{BSR}	0.00087
BSL_{MAX}	0.4777
Km_{BSL}	0.0087
$CSQN_{MAX,CSR}$	2.88
$CSQN_{MAX,JSR}$	1.2
Km_{CSQN}	0.8
$CMDN_{MAX,i}$	0.1125
$CMDN_{MAX,sl}$	1.25e-2
Km_{CMDN}	0.00238
$TRPN_{MAX,I}$	3.15e-2
$TRPN_{MAX,SL}$	3.5e-3
Km_{TRPN}	0.0005

Voltage

$$C_m \frac{dV}{dt} = -(I_{ion} + I_{stim})$$

$$I_{ion} = I_{Na} + I_{NaL} + I_{to} + I_{sus} + I_{CaL} + I_{caT} + I_{CaNa} + I_{CaK} + I_{Kr} + I_{Ks} + I_f + I_{K1} + I_{NaCa} + I_{NaK} + I_{Nab} + I_{Cab} + I_{pCa} + I_{Stim}$$

Fast-Sodium Current (I_{Na})

from (Dutta et al. 2017; Passini et al. 2016)

$$m_\infty = \frac{1}{1 + e^{-\left(\frac{V+48.6264}{9.871}\right)}}$$

$$\tau_m = \frac{1}{6.755 \cdot e^{\left(\frac{V+11.64}{34.77}\right)} + 8.552 \cdot e^{-\left(\frac{V+77.42}{5.955}\right)}}$$

$$h_\infty = \frac{1}{1 + e^{\left(\frac{V+78.5}{6.22}\right)}}$$

$$\tau_{h,fast} = \frac{1}{3.686 \cdot 10^{-6} \cdot e^{-\left(\frac{V+3.8875}{7.8579}\right)} + 16 \cdot e^{\left(\frac{V-0.4963}{9.1843}\right)}}$$

$$\tau_{h,slow} = \frac{1}{0.009764 \cdot e^{-\left(\frac{V+17.95}{28.05}\right)} + 0.3343 \cdot e^{\left(\frac{V+5.730}{56.66}\right)}}$$

$A_{h,fast} = 0.99,$ $\quad A_{h,slow} = 1 - A_{h,fast}$

$$h = A_{h,fast} \cdot h_{fast} + A_{h,slow} \cdot h_{slow}$$

$$j_\infty = h_\infty$$

$$\tau_j = 4.8590 + \frac{1}{0.8628 \cdot e^{-\left(\frac{V+166.7}{7.6}\right)} + 1.1096 \cdot e^{\left(\frac{V+6.2719}{9.0358}\right)}}$$

$$h_{CaMK,\infty} = \frac{1}{1 + e^{\left(\frac{V+84.7}{6.22}\right)}}$$

$$\tau_{h,CaMK,slow} = 3 \cdot \tau_{h,slow}$$

$A_{h,CaMK,fast} = A_{h,fast}$ $\quad A_{h,CaMK,slow} = A_{h,slow}$

$$h_{CaMK,\infty} = h_{fast}$$

$$h_{CaMK} = A_{h,CaMK,fast} \cdot h_{CaMK,fast} + A_{h,CaMK,slow} \cdot h_{CaMK,slow}$$

$$j_{CaMK,\infty} = j_\infty$$

$$\tau_{j,CaMK} = 1.46 \cdot \tau_j$$

$K_{m,CaMK} = 0.15,$ $\quad \emptyset_{INa,CaMK} = \frac{1}{1+\frac{K_{m,CaMK}}{K_{m,CaMK,active}}}$

$$I_{Na,fast} = G_{Na} \cdot (V - E_{Na}) \cdot m^3 \cdot \left((1 - \emptyset_{INa,CaMK}) \cdot h \cdot j + \emptyset_{INa,CaMK} \cdot h_{CaMK} \cdot j_{CaMK}\right)$$

Late-Sodium Current (I_{NaL})

from (O'Hara et al. 2011)

$$m_{L,\infty} = \frac{1}{1+e^{-\left(\frac{V+42.85}{5.264}\right)}}$$

$$\tau_{m,L} = \tau_m$$

$$h_{L,\infty} = \frac{1}{1+e^{\left(\frac{V+87.61}{7.488}\right)}}$$

$$\tau_{h,L} = 200\ \text{ms}$$

$$h_{L,CaMK,\infty} = \frac{1}{1+e^{\left(\frac{V+93.81}{7.488}\right)}}$$

$$\tau_{h,LCaMK} = 3 \cdot \tau_{h,L}$$

$$K_{m,CaMK} = 0.15, \qquad \emptyset_{INaL,CaMK} = \frac{1}{1+\frac{K_{m,CaMK}}{K_{m,CaMK,active}}}$$

$$I_{Na,late} = G_{NaL} \cdot (V - E_{Na}) \cdot m_L \cdot \left((1 - \emptyset_{INaL,CaMK}) \cdot h_L + \emptyset_{INaL,CaMK} \cdot h_{L,CaMK}\right)$$

L-type Calcium Current (I_{CaL})

from (O'Hara et al. 2011), modified as described in 1.1

$$d = \frac{1}{1+e^{-\left(\frac{V+7.24}{4.23}\right)}}$$

$$\tau_d = 0.6 + \frac{1}{e^{-0.05\cdot(V+6)} + e^{-0.09\cdot(V+14)}}$$

$$f_\infty = \frac{1}{1+e^{\left(\frac{V+22.88}{3.696}\right)}}$$

$$\tau_{f,fast} = 7 + \frac{1}{0.0045 \cdot e^{-\left(\frac{V+35.19}{10}\right)} + 0.0045 \cdot e^{\left(\frac{V+35.19}{10}\right)}}$$

$$\tau_{f,slow} = 1000 + \frac{1}{0.000035 \cdot e^{-\left(\frac{V+20.19}{4}\right)} + 0.000035 \cdot e^{\left(\frac{V+20.19}{4}\right)}}$$

$$A_{f,fast} = 0.6, \qquad A_{f,slow} = 1 - A_{f,fast}$$

$$f = A_{f,fast} \cdot f_{fast} + A_{f,slow} \cdot f_{slow}$$

$$f_{Ca,\infty} = f_\infty$$

$$\tau_{f,Ca,fast} = 7 + \frac{1}{0.04 \cdot e^{-\left(\frac{V-11.19}{7}\right)} + 0.04 \cdot e^{\left(\frac{V-11.19}{7}\right)}}$$

$$\tau_{f,Ca,slow} = 100 + \frac{1}{0.00012 \cdot e^{-\left(\frac{V+20}{3}\right)} + 0.00012 \cdot e^{\left(\frac{V+20}{7}\right)}}$$

$$A_{f,\,Ca,\,fast} = 0.3 + \frac{0.6}{1+e^{\left(\frac{V-10}{10}\right)}}, \qquad A_{f,Ca,slow} = 1 - A_{f,Ca,fast}$$

$$f_{Ca} = A_{f,Ca,fast} \cdot f_{Ca,fast} + A_{f,Ca,slow} \cdot f_{Ca,slow}$$

$$j_{Ca,\infty} = f_{Ca,\infty}$$

$$\tau_{j,Ca} = 75$$

$$f_{CaMK,\infty} = f_{\infty}$$

$$\tau_{f,CaMK,fast} = 2.5 \cdot \tau_{f,fast}$$

$$A_{f,CaMK,fast} = A_{f,fast}, \qquad A_{f,CaMK,slow} = A_{f,slow}$$

$$f_{CaMK,slow} = f_{slow}$$

$$f_{CaMK} = A_{f,CaMK,fast} \cdot f_{CaMK,fast} + A_{f,CaMK,slow} \cdot f_{CaMK,slow}$$

$$f_{Ca,CaMK,\infty} = f_{\infty}$$

$$\tau_{f,Ca,CaMK,fast} = 2.5 \cdot \tau_{f,Ca,fast}$$

$$A_{f,Ca,CaMK,fast} = A_{f,Ca,fast}, \qquad A_{f,Ca,CaMK,slow} = A_{f,Ca,slow}$$

$$f_{Ca,CaMK,slow} = f_{Ca,slow}$$

$$f_{Ca,CaMK} = A_{f,Ca,CaMK,fast} \cdot f_{Ca,CaMK,fast} + A_{f,Ca,CaMK,slow} \cdot f_{Ca,CaMK,slow}$$

$$K_{m,n} = 0.002, \quad K_{+2,n} = 1000, \qquad K_{-2,n} = j_{Ca}$$

$$\alpha_n = \frac{1}{\frac{K_{+2,n}}{K_{-2,n}} + \left(1 + e^{\left(\frac{K_{m,n}}{[Ca^{2+}]_{sl}}\right)}\right)^4}$$

$$\gamma_{Cai} = 1, \qquad \gamma_{Cao} = 0.341, \quad z_{Ca} = 2$$

$$\Psi_{Ca} = z_{Ca}^2 \cdot \frac{VF^2}{RT} \cdot \frac{\gamma_{Cai} \cdot [Ca^{2+}]_{SS} \cdot e^{\frac{z_{Ca}VF}{RT}} - \gamma_{Cao} \cdot [Ca^{2+}]_o}{e^{\frac{z_{Ca}VF}{RT}} - 1.0}$$

$$\overline{I_{CaL}} = G_{CaL} \cdot \Psi_{Ca}$$

$$P_{CaNa} = 0.00125 \cdot P_{Ca}, \qquad \gamma_{Nai} = 0.75, \quad \gamma_{Nao} = 0.75, \quad z_{Na} = 1$$

$$\Psi_{CaNa} = z_{Na}^2 \cdot \frac{VF^2}{RT} \cdot \frac{\gamma_{Nai} \cdot [Na^{+}]_{SS} \cdot e^{\frac{z_{Na}VF}{RT}} - \gamma_{Nao} \cdot [Na^{+}]_o}{e^{\frac{z_{Na}VF}{RT}} - 1.0}$$

$$\overline{I_{CaNa}} = P_{CaNa} \cdot \Psi_{CaNa}$$

$P_{CaK} = 3.574 \cdot 10^{-4} \cdot P_{Ca}$, $\gamma_{Ki} = 0.75,$ $\gamma_{Ko} = 0.75,$ $z_K = 1$

$$\Psi_{CaK} = z_K^2 \cdot \frac{VF^2}{RT} \cdot \frac{\gamma_{Ki} \cdot [K^+]_{SS} \cdot e^{\frac{z_K VF}{RT}} - \gamma_{Ko} \cdot [K^+]_o}{e^{\frac{z_K VF}{RT}} - 1.0}$$

$$\overline{I_{CaK}} = P_{CaK} \cdot \Psi_{CaK}$$

$$P_{Ca,CaMK} = 1.1 \cdot G_{CaL}$$

$$\overline{I_{CaL,CaMK}} = P_{Ca,CaMK} \cdot \Psi_{Ca}$$

$$P_{CaNa,CaMK} = 0.00125 \cdot P_{Ca,CaMk}$$

$$\overline{I_{CaNa,CaMK}} = P_{CaNa,CaMK} \cdot \Psi_{CaNa}$$

$$P_{CaK,CAMK} = 3.574 \cdot 10^{-4} \cdot P_{Ca,CaMK}$$

$$\overline{I_{CaK,CaMK}} = P_{CaK,CaMK} \cdot \Psi_{CaK}$$

$$K_{m,CaMK} = 0.15, \qquad \emptyset_{ICaL,CaMK} = \frac{1}{1+\frac{K_{m,CaMK}}{CaMK_{active}}}$$

$$I_{CaL} = \overline{I_{CaL}} \cdot d \cdot \left(1 - \emptyset_{ICaL,CaMK}\right) \cdot (f \cdot (1-n) + f_{Ca} \cdot n \cdot j_{Ca}) + \overline{I_{CaL,CaMK}} \cdot d \cdot \emptyset_{ICaL,CaMK} \cdot \left(f_{CaMK} \cdot (1-n) + f_{Ca,CaMK} \cdot n \cdot j_{Ca}\right)$$

$$I_{CaNa} = \overline{I_{CaNa}} \cdot d \cdot \left(1 - \emptyset_{ICaL,CaMK}\right) \cdot (f \cdot (1-n) + f_{Ca} \cdot n \cdot j_{Ca}) + \overline{I_{CaNa,CaMK}} \cdot d \cdot \emptyset_{ICaL,CaMK} \cdot \left(f_{CaMK} \cdot (1-n) + f_{Ca,CaMK} \cdot n \cdot j_{Ca}\right)$$

$$I_{CaK} = \overline{I_{CaK}} \cdot d \cdot \left(1 - \emptyset_{ICaL,CaMK}\right) \cdot (f \cdot (1-n) + f_{Ca} \cdot n \cdot j_{Ca}) + \overline{I_{CaK,CaMK}} \cdot d \cdot \emptyset_{ICaL,CaMK} \cdot \left(f_{CaMK} \cdot (1-n) + f_{Ca,CaMK} \cdot n \cdot j_{Ca}\right)$$

T-type Calcium Current (I_{CaT})

From (Pan and Rudy 2011)

$$b_\infty = \frac{1}{1 + e^{-(\frac{V+30}{7})}}$$

$$\tau_b = \frac{1}{1.068 \cdot e^{-\left(\frac{V-16.3}{30}\right)} + 1.068 \cdot e^{\left(\frac{V-16.3}{30}\right)}}$$

$$g_\infty = \frac{1}{1 + e^{(\frac{V+61}{5})}}$$

$$\tau_b = \frac{1}{0.015 \cdot e^{-\left(\frac{V-71.7}{83.3}\right)} + 0.015 \cdot e^{\left(\frac{V+71.7}{15.4}\right)}}$$

$$I_{CaT} = G_{CaT} \cdot b \cdot g \cdot (V - E_{Ca})$$

Transient Outward Current (I_{to})

Data from (Han et al. 2002)

$$a_\infty = \frac{1}{1+e^{\left(\frac{20-V}{13}\right)}}$$

$$\tau_a = \frac{1.0515}{\left(\frac{1}{1.2\cdot\left(1+\exp\left(-\frac{V-18.41}{29.38}\right)\right)}+\frac{3.5}{1+\exp\left(\frac{V+100}{29.38}\right)}\right)}$$

$$i_{1,\infty} = \frac{1}{1+e^{\left(\frac{V+27}{13}\right)}}$$

$$i_{2,\infty} = i_{1.\infty}$$

$$\tau_{i,S} = 43 + \frac{1}{0.001416\cdot\exp\left(-\frac{V+96.52}{59.05}\right)+1.7\cdot 10^{-8}\cdot\exp\left(\frac{V+114.1}{8.079}\right)}$$

$$\tau_{i,F} = 6.16 + \frac{1}{0.39\cdot\exp\left(-\frac{V+100}{100}\right)+0.08\cdot\exp\left(\frac{V-8}{8.59}\right)}$$

$$I_{to}=g_{to}\cdot a\cdot i_S\cdot i_F\cdot g\cdot(V - E_k)$$

Sustained Potassium Current (I_{sus})

Data from (Han et al. 2002)

$$a_{sus} = \frac{1}{1+e^{-\left(\frac{V-12}{16}\right)}}$$

$$i_{sus} = g_{sus}\cdot a_{sus}\cdot(V-E_K)$$

Rapid Delayed Rectifier Potassium Current (I_{Kr})

from (O’Hara et al. 2011), modified as described in 1.1

$$x_{r\infty} = \frac{1}{1+e^{-\left(\frac{V+8.337}{6.789}\right)}}$$

$$\tau_{xr,fast} = 12.98 + \frac{1}{0.3652\cdot e^{\left(\frac{V-14.06}{3.869}\right)}+4.123\cdot 10^{-5}\cdot e^{-\left(\frac{V-30.18}{20.38}\right)}}$$

$$\tau_{xr,slow} = 1.865 + \frac{1}{0.06629\cdot e^{\left(\frac{V-19.7}{7.355}\right)}+1.128\cdot 10^{-5}\cdot e^{-\left(\frac{V-12.54}{25.94}\right)}}$$

$$A_{xr,fast} = \frac{1}{1+e^{\left(\frac{V+54.81}{38.21}\right)}}, \qquad A_{xr,slow} = 1 - A_{xr,fast}$$

$$x_r = A_{xr,fast}\cdot x_{r,fast} + A_{xr,slow}\cdot x_{r,slow}$$

$$R_{Kr} = \frac{1}{\left(1+e^{\left(\frac{V+55}{24}\right)}\right)\cdot\left(1+e^{\left(\frac{V-10}{9.6}\right)}\right)}$$

$$i_{Kr} = g_{Kr}\cdot\sqrt{\frac{[K^+]_o}{5.4}}\cdot x_r\cdot R_{Kr}\cdot(V-E_K)$$

Slow Delayed Rectifier Potassium Current (I_{Ks})

from (O'Hara et al. 2011)

$$x_{s1,\infty} = \frac{1}{1 + e^{-\left(\frac{V+11.6}{8.932}\right)}}$$

$$\tau_{x,s1} = 817.3 + \frac{1}{0.001292 \cdot e^{-\left(\frac{V+210}{230}\right)} + 2.326 \cdot 10^{-4} \cdot e^{\left(\frac{V+48.28}{17.8}\right)}}$$

$$x_{s2,\infty} = x_{s1,\infty}$$

$$\tau_{x,s2} = \frac{1}{0.01 \cdot e^{\left(\frac{V-50}{20}\right)} + 0.0193 \cdot e^{-\left(\frac{V+66.54}{31}\right)}}$$

$$i_{Ks} = g_{Ks} \cdot \left(1 + \frac{0.6}{1 + \left(\frac{3.8 \cdot 10^{-5}}{[Ca^{2+}]_{sl}}\right)^{1.4}}\right) \cdot x_{s1} \cdot x_{s2} \cdot (V - E_{Ks})$$

Hyperpolarization-activated Current (I_f)

from (Pan and Rudy 2011)

$$y_\infty = \frac{1}{1 + e^{\left(\frac{V+87}{9.5}\right)}}$$

$$\tau_y = \frac{2000}{\exp\left(-\frac{V + 132}{10}\right) + \exp\left(\frac{V + 57}{60}\right)}$$

$$i_{f,K} = g_{f,K} \cdot y \cdot (V - E_K)$$

$$i_{f,Na} = g_{f,Na} \cdot y \cdot (V - E_{Na})$$

$$i_f = i_{f,K} + i_{f,Na}$$

Inward Rectifier Potassium Current (I_{K1})

Data from (Han et al. 2002)

$$x_{K1,\infty} = \frac{1}{1 + e^{-\left(\frac{V+2.5538 \cdot [K^+]_o + 144.59}{1.5692 \cdot [K^+]_o + 3.8115}\right)}}$$

$$\tau_{x,K1} = \frac{122.2}{e^{-\left(\frac{V+127.2}{20.36}\right)} + e^{\left(\frac{V+236.8}{69.33}\right)}}$$

$$R_{K1} = \frac{1}{1 + e^{\left(\frac{V+116-5.5 \cdot [K^+]_o}{11}\right)}}$$

$$i_{K1} = g_{K1} \cdot \sqrt{\frac{[K^+]_o}{5.4}} \cdot x_{K1} \cdot R_{K1} \cdot (V - E_K)$$

Sodium-Calcium Exchange Current (I_{NaCa})

from (O'Hara et al. 2011)

For, Y ∈ {i, ss}:

$K_{Na1} = 15\ \text{mM}$, $K_{Na2} = 5\ \text{mM}$, $K_{Na3} = 88.12\ \text{mM}$, $K_{asymm} = 12.5$

$\omega_{Na} = 6 \cdot 10^4\ \text{Hz}$, $\omega_{Ca} = 6 \cdot 10^4\ \text{Hz}$, $\omega_{NaCa} = 5 \cdot 10^3\ \text{Hz}$

$k_{Ca,on} = 1.5 \cdot 10^6\ \frac{\text{mM}}{\text{ms}}$, $k_{Ca,off} = 5 \cdot 10^3\ \text{Hz}$

$q_{Na} = 0.5224$, $q_{Ca} = 0.1670$

$h_{Ca} = \exp\left(\frac{q_{Ca}VF}{RT}\right)$, $h_{Na} = \exp\left(\frac{q_{Na}VF}{RT}\right)$,

$$h_1 = 1 + \frac{[Na^+]_Y}{k_{Na3}}(1 + h_{Na})$$

$$h_2 = \frac{[Na^+]_Y \cdot h_{Na}}{k_{Na3} \cdot h_1}$$

$$h_3 = \frac{1}{h_1}$$

$$h_4 = 1 + \frac{[Na^+]_Y}{k_{Na1}}\left(1 + \frac{[Na^+]_Y}{k_{Na2}}\right)$$

$$h_5 = \frac{[Na^+]_Y{}^2}{k_4 \cdot k_{Na1} \cdot k_{Na2}}$$

$$h_6 = \frac{1}{h_4}$$

$$h_7 = 1 + \frac{[Na^+]_o}{k_{Na3}}\left(1 + \frac{1}{h_{Na}}\right)$$

$$h_8 = \frac{[Na^+]_o}{k_{Na3} \cdot h_{Na} \cdot h_7}$$

$$h_9 = \frac{1}{h_7}$$

$$h_{10} = k_{asymm} + 1 + \frac{[Na^+]_o}{k_{Na1}}\left(1 + \frac{[Na^+]_o}{k_{Na2}}\right)$$

$$h_{11} = \frac{[Na^+]_o{}^2}{h_{10} \cdot k_{Na1} \cdot k_{Na2}}$$

$$h_{12} = \frac{1}{h_{10}}$$

$$k_1 = h_{12} \cdot [Ca^{2+}]_o \cdot k_{Ca,on}$$

$$k_2 = k_{Ca,off}$$

$$k_3{}' = h_9 \cdot \omega_{Ca}$$

$$k_3{}'' = h_8 \cdot \omega_{NaCa}$$

$$k_3 = k_3{}' + k_3{}''$$

$$k_4{}' = \frac{h_3 \cdot \omega_{Ca}}{h_{Ca}}$$

$$k_4{}'' = h_2 \cdot \omega_{NaCa}$$

$$k_4 = k_4{}' + k_4{}''$$

$$k_5 = k_{Ca,off}$$

$k_6 = h_6 \cdot [Ca^{2+}]_Y \cdot k_{Ca,on}$
$k_7 = h_5 \cdot h_2 \cdot \omega_{Na}$
$k_8 = h_8 \cdot h_{11} \cdot \omega_{Na}$
$x_1 = k_2 \cdot k_4 \cdot (k_7 + k_6) + k_5 \cdot k_7 \cdot (k_2 + k_3)$
$x_2 = k_1 \cdot k_7 \cdot (k_4 + k_5) + k_4 \cdot k_6 \cdot (k_1 + k_8)$
$x_3 = k_1 \cdot k_3 \cdot (k_7 + k_6) + k_8 \cdot k_6 \cdot (k_2 + k_3)$
$x_4 = k_2 \cdot k_8 \cdot (k_4 + k_5) + k_3 \cdot k_5 \cdot (k_1 + k_8)$

$$E_1 = \frac{x_1}{x_1 + x_2 + x_3 + x_4}$$
$$E_2 = \frac{x_2}{x_1 + x_2 + x_3 + x_4}$$
$$E_3 = \frac{x_3}{x_1 + x_2 + x_3 + x_4}$$
$$E_4 = \frac{x_4}{x_1 + x_2 + x_3 + x_4}$$

$K_{mCaAct} = 150 \cdot 10^{-6}$ mM

$$allo_Y = \frac{1}{1 + \left(\frac{K_{mCaACt}}{[Ca^{2+}]_Y}\right)^2}$$

$J_{NaCa,Na,Y} = 3 \cdot (E_4 \cdot k_7 - E_1 \cdot k_8) + E_3 \cdot k_4'' - E_2 \cdot k_3''$
$J_{NaCa,Ca,Y} = E_2 \cdot k_2 - E_1 \cdot k_1$
$z_{Na} = 1$; $z_{Ca} = 2$;
$I_{NaCa,i} = G_{NaCa} \cdot 0.8 \cdot allo_i \cdot (z_{Na} \cdot J_{NaCa,Na,i} + z_{Ca} \cdot J_{NaCa,Ca,i})$
$I_{NaCa,ss} = G_{NaCa} \cdot 0.2 \cdot allo_{ss} \cdot (z_{Na} \cdot J_{NaCa,Na,ss} + z_{Ca} \cdot J_{NaCa,Ca,ss})$
$I_{NaCa} = I_{NaCa,i} + I_{NaCa,ss}$

Sodium-Potassium ATPase Current (I_{NaK})

from (O'Hara et al. 2011)

$k_1^+ = 949.5$ Hz ; $k_1^- = 182.4$ mM^{-1} ; $k_2^+ = 687.2$ Hz ; $k_2^- = 39.4$ Hz
$k_3^+ = 1899$ Hz ; $k_3^- = 79300$ Hz $\cdot$ mM^{-2} ; $k_4^+ = 639.0$ Hz ; $k_4^- = 40$ Hz
$K_{Nai}^0 = 9.073$ mM ; $K_{Nao}^0 = 27.78$ mM
$\Delta = -01550$
$K_{Nai} = K_{Nai}^0 \cdot \exp\left(\frac{\Delta \cdot V \cdot F}{3 \cdot R \cdot T}\right)$; $K_{Nao} = K_{Nao}^0 \cdot \exp\left(\frac{(1-\Delta) \cdot V \cdot F}{3 \cdot R \cdot T}\right)$
$K_{Ki} = 0.5$ mM ; $K_{Ko} = 0.3582$ mM
$[MgADP] = 0.05$; $[MgATP] = 9.8$
$[K_{MgATP}] = 1.698 \cdot 10^{-7}$ mM
$[H^+] = 10^{-7}$ mM
$[\Sigma P] = 4.2$ mM
$K_{H,P} = 1.698 \cdot 10^{-7}$ mM, $K_{Na,P} = 224$ mM, $K_{K,P} = 292$ mM

$$[P] = \frac{[\Sigma P]}{\left(1 + \frac{[H^+]}{K_{H,P}} + \frac{[Na^+]_i}{K_{Na,P}} + \frac{[K^+]_i}{K_{K,P}}\right)}$$

$$\alpha_1 = \frac{k_1^+ \cdot \left(\frac{[Na^+]_i}{K_{Nai}}\right)^3}{\left(1 + \frac{[Na^+]_i}{K_{Nai}}\right)^3 + \left(1 + \frac{[K^+]_i}{K_{Ki}}\right)^2 - 1}$$

$$\beta_2 = k_1^{\,-} \cdot [MgADP]$$

$$\alpha_2 = k_2^+$$

$$\beta_2 = \frac{k_2^- \cdot \left(\frac{[Na^+]_o}{K_{Nao}}\right)^3}{\left(1 + \frac{[Na^+]_o}{K_{Nao}}\right)^3 + \left(1 + \frac{[K^+]_o}{K_{Ko}}\right)^2 - 1}$$

$$\alpha_3 = \frac{k_3^+ \cdot \left(\frac{[K^+]_o}{K_{Ko}}\right)^2}{\left(1 + \frac{[Na^+]_o}{K_{Nao}}\right)^3 + \left(1 + \frac{[K^+]_o}{K_{Ko}}\right)^2 - 1}$$

$$\beta_3 = \frac{k_3^- \cdot [P] \cdot [H^+]}{1 + \frac{[MgATP]}{KMgATP}}$$

$$\alpha_4 = \frac{k_4^+ \cdot [P] \cdot [H^+]}{1 + \frac{[MgATP]}{KMgATP}}$$

$$\beta_4 = \frac{k_4^- \cdot \left(\frac{[K^+]_i}{K_{Ki}}\right)^3}{\left(1 + \frac{[Na^+]_i}{K_{Nai}}\right)^3 + \left(1 + \frac{[K^+]_i}{K_{Ki}}\right)^2 - 1}$$

$$x_1 = \alpha_1 \cdot \alpha_2 \cdot \alpha_4 + \beta_2 \cdot \beta_3 \cdot \beta_4 + \alpha_2 \cdot \beta_3 \cdot \beta_4 + \beta_3 \cdot \alpha_1 \cdot \alpha_2$$

$$x_2 = \alpha_1 \cdot \alpha_2 \cdot \alpha_3 + \beta_1 \cdot \beta_2 \cdot \beta_4 + \alpha_3 \cdot \beta_1 \cdot \beta_4 + \beta_4 \cdot \alpha_2 \cdot \alpha_3$$

$$x_3 = \alpha_2 \cdot \alpha_3 \cdot \alpha_4 + \beta_1 \cdot \beta_2 \cdot \beta_3 + \alpha_4 \cdot \beta_2 \cdot \beta_1 + \beta_1 \cdot \alpha_3 \cdot \alpha_4$$

$$x_4 = \alpha_1 \cdot \alpha_3 \cdot \alpha_4 + \beta_2 \cdot \beta_3 \cdot \beta_4 + \alpha_1 \cdot \beta_2 \cdot \beta_3 + \beta_2 \cdot \alpha_1 \cdot \alpha_4$$

$$E_1 = \frac{x_1}{x_1+x_2+x_3+x_4}$$

$$E_2 = \frac{x_2}{x_1+x_2+x_3+x_4}$$

$$E_3 = \frac{x_3}{x_1+x_2+x_3+x_4}$$

$$E_4 = \frac{x_4}{x_1+x_2+x_3+x_4}$$

$$z_{Na} = 1\,;\; z_k = 1\,;$$

$$J_{NaK,Na} = 3\,(E_1 \cdot \alpha_3 - E_2 \cdot \beta_3)$$

$$J_{NaK,K} = 2\,(E_4 \cdot \beta_1 - E_3 \cdot \alpha_1)$$

$$I_{NaK} = G_{NaK} \cdot (z_{Na} \cdot J_{NaK,Na} + z_K \cdot J_{NaK,K})$$

***Background** currents: I_{Nab}, I_{Cab}, I_{pCa}*

from (O’Hara et al. 2011)

$$P_{Nab} = 3.75 \cdot 10^{-10}\ cm/s\,;\quad z_{Na} = 1;$$

$$I_{Nab} = P_{Nab} \cdot {z_{Na}}^2 \cdot \frac{VF^2}{RT} \cdot \frac{[Na^+]_i \cdot \exp\left(\frac{VFz_{Na}}{RT}\right) - [Na^+]_o}{\exp\left(\frac{VFz_{Na}}{RT}\right) - 1}$$

$P_{Cab} = 2.5 \cdot 10^{-8}$ cm/s; $\gamma_{Cai} = 1$; $\gamma_{Cao} = 0.341$; $z_{Ca} = 2$;

$$I_{Cab} = P_{Cab} \cdot {z_{Ca}}^2 \cdot \frac{VF^2}{RT} \cdot \frac{\gamma_{Cai} \cdot [Ca^{2+}]_i \cdot \exp\left(\frac{VFz_{Ca}}{RT}\right) - \gamma_{Cao} \cdot [Ca^{2+}]_o}{\exp\left(\frac{VFz_{Ca}}{RT}\right) - 1}$$

$G_{pCa} = 0.0005$ mS/uF;

$$I_{pCa} = G_{pCa} \cdot \frac{[Ca^{2+}]_{sl}}{0.0005 + [Ca^{2+}]_{sl}}$$

Calcium/Calmodulin-Dependent Protein Kinase (CaMK)

from (O'Hara et al. 2011)

$\alpha_{CaMK} = 0.05\ ms^{-1}$; $\beta_{CaMK} = 0.00068\ ms^{-1}$;

$CaMK_0 = 0.05$; $K_{mCaM} = 0.0015$ mM

$$CaMK_{bound} = CaMK_0 \cdot \frac{1 - CaMK_{trap}}{1 + \frac{K_{mCaM}}{[Ca^{2+}]_{ss}}}$$

$$CaMK_{active} = CaMK_{bound} + CaMK_{trap}$$

$$\frac{dCaMK_{trap}}{dt} = \alpha_{CaMK} \cdot CaMK_{bound} \cdot (CaMK_{bound} + CaMK_{trap}) - \beta_{CaMK} \cdot CaMK_{trap}$$

Sarcoplasmic Reticulum Ca^{2+} Fluxes

from in (Pan and Rudy 2011)

- ***RyR3***

$$Rel_{RyR3} = -\left(I_{CaL} \cdot \frac{A_{Cap}}{V_{SS} \cdot 2 \cdot F} - (J_{RyR3} + J_{IP3R}\right) \cdot \frac{V_{JSR}}{V_{SS}} + J_{diff1}$$

$$\tau_{RyR3} = \frac{2 \cdot (1 + \frac{1}{1 + \left(\frac{0.28}{[CaMK_{active}]}\right)^8}}{1 + \frac{0.0123}{[Ca^{2+}]_{JSR}}}$$

If ($Rel_{RyR3} > 0$)

$$RyR3_\infty = \frac{15 \cdot Rel_{RyR3} \cdot (1 + \frac{1}{1 + \left(\frac{0.28}{[CaMK_{active}]}\right)^8}}{1 + \left(\frac{1}{[Ca^{2+}]_{JSR}}\right)^8}$$

else

$$RyR3_\infty = 0$$

$$\frac{dJ_{RyR3}}{dt} = \frac{RyR3_\infty - J_{RyR3}}{\tau_{RyR3}}$$

- ***RyR2***

$$\mathrm{Rel_{RyR2}} = -J_{SERCA} \cdot \frac{V_{NSR}}{V_{Myo}} + J_{diff2} \cdot \frac{V_{SL}}{V_{Myo}} + J_{RyR2} \cdot \frac{V_{CSR}}{V_{Myo}}$$

$$\tau_{RyR2} = \frac{6 \cdot (1 + \frac{1}{1 + \left(\frac{0.28}{[CaMK_{active}]}\right)^8}}{1 + \frac{0.0123}{[Ca^{2+}]_{CSR}}}$$

If $(\mathrm{Rel_{RyR2}} > 0)$

$$\mathrm{RyR2_\infty} = \frac{91 \cdot \mathrm{Rel_{RyR2}} \cdot (1 + \frac{1}{1 + \left(\frac{0.28}{[CaMK_{active}]}\right)^8}}{1 + \left(\frac{1}{[Ca^{2+}]_{CSR}}\right)^8}$$

else

$$\mathrm{RyR2_\infty} = 0$$

$$\frac{\mathrm{dJ}_{RyR2}}{\mathrm{dt}} = \frac{\mathrm{RyR2_\infty} - \mathrm{J_{RyR2}}}{\tau_{RyR2}}$$

- ***IP_3R Ca^{2+} release:***

$\mathrm{k_0} = 96000\,\mathrm{mM^{-1}s^{-1}};\ \mathrm{k_{0a}} = 9.6\,\mathrm{s^{-1}};\ \mathrm{k_1} = 150000\,\mathrm{mM^{-1}s^{-1}};\ \mathrm{k_{1a}} = 16.5\,\mathrm{s^{-1}}$

$\mathrm{k_2} = 1800\,\mathrm{mM^{-1}s^{-1}};\ \mathrm{k_{2a}} = 0.21\mathrm{s^{-1}};\ \tau_{\mathrm{IP3R}} = 3.7\mathrm{s^{-1}}$

$[IP_3]$ = 0.001 mM/L;

$$\frac{du_{IP3R}}{dt} = [Ca^{2+}]_{SS} \cdot \mathrm{k_2} \cdot (1 - \mathrm{u}_{IP3R}) - \mathrm{k_{2a}} \cdot \mathrm{u}_{IP3R}$$

$$\mathrm{J_{IP3R}} = 10.92 \cdot \frac{\tau_{\mathrm{IP3R}} \cdot [\mathrm{IP3}] \cdot [Ca^{2+}]_{SS} \cdot (1 - \mathrm{u}_{IP3R})}{\left(1 + \frac{[IP3] \cdot k_0}{k_{0a}}\right) \cdot \left(1 + [Ca^{2+}]_{SS} \frac{k_1}{k_{1a}}\right)} \left([Ca^{2+}]_{JSR} - [Ca^{2+}]_{SS}\right)$$

- ***Ca^{2+} uptake via SERCA:***

$\Delta K_{0m,PLB} = 0.00017 \frac{mM}{L};$ $\Delta \mathrm{J}_{0,SERCA,CAMK} = 0.75;$ $\mathrm{K}_{m,CAMK} = 0.15;$

$\mathrm{J}_{0,SERCA,1} = 0.0002 \frac{mM}{L}/ms;$ $\mathrm{J}_{0,SERCA,2} = 0.0026 \frac{mM}{L}/ms;$ $\mathrm{K}_{m,SERCA} = 0.00028 \frac{mM}{L};$

$$\overline{\mathrm{NSR}} = 15 \frac{mM}{L}$$

$$\Delta K_{m,PLB} = \Delta K_{0m,PLB} \cdot \frac{CAMK_{active}}{\mathrm{K}_{m,CAMK} + CAMK_{active}}$$

$$\Delta J_{SERCA,CAMK} = \Delta J_{0,SERCA,CAMK} \cdot \frac{CAMK_{active}}{\mathrm{K}_{m,CAMK} + CAMK_{active}}$$

$$\mathrm{J}_{SERCA,1} = \mathrm{J}_{0,SERCA,1} \cdot \frac{(1 + \Delta J_{SERCA,CAMK})}{1 + \frac{K_{m,SERCA} - \Delta K_{m,PLB}}{[Ca^{2+}]_{SL}}} - 0.0042 \cdot \frac{[Ca^{2+}]_{NSR}}{\overline{NSR}}$$

$$J_{SERCA,2} = J_{0,SERCA,2} \cdot \frac{\left(1 + \Delta J_{SERCA,CAMK}\right)}{1 + \frac{K_{m,SERCA} - \Delta K_{m,PLB}}{[Ca^{2+}]_i}} - 0.00105 \cdot \frac{[Ca^{2+}]_{NSR}}{\overline{\mathrm{NSR}}}$$

Diffusion Fluxes

$$\tau_{tr} = 120\ ms$$

$$J_{tr,1} = \frac{([Ca^{2+}]_{NSR} - [Ca^{2+}]_{JSR})}{\tau_{tr}}; \qquad J_{tr,2} = \frac{([Ca^{2+}]_{NSR} - [Ca^{2+}]_{CSR})}{\tau_{tr}}$$

$$\tau_{diff1} = 0.2\ ms; \qquad \tau_{diff2} = 12\ ms$$

$$J_{diff1,Ca} = \frac{([Ca^{2+}]_{SS} - [Ca^{2+}]_{SL})}{\tau_{diff1}}; \qquad J_{diff2,Ca} = \frac{([Ca^{2+}]_{SL} - [Ca^{2+}]_{i})}{\tau_{diff2}}$$

$$J_{diff1,Na} = \frac{([Na^{+}]_{SS} - [Na^{+}]_{SL})}{\tau_{diff1}}; \qquad J_{diff2,Na} = \frac{([Na^{+}]_{SL} - [Na^{+}]_{i})}{\tau_{diff2}}$$

$$J_{diff1,K} = \frac{([K^{+}]_{SS} - [K^{+}]_{SL})}{\tau_{diff1}}; \qquad J_{diff2,K} = \frac{([K^{+}]_{SL} - [K^{+}]_{i})}{\tau_{diff2}}$$

Ionic Concentrations

- ***$[Ca^{2+}]_{SS}$***

$$\beta_{PCS} = \frac{1}{1 + \overline{\mathrm{BSR}} \cdot \frac{K_{m,BSR}}{([Ca^{2+}]_{SS} + K_{m,BSR})} + \overline{\mathrm{BSL}} \cdot \frac{K_{m,BSL}}{([Ca^{2+}]_{Ss} + K_{m,BSL})}}$$

$$\frac{d\left[Ca^{2+}\right]_{SS}}{dt} = \beta_{PCS} \cdot (-(I_{CaL} - 2 \cdot I_{NaCa,SS}) \cdot \frac{A_{Cap}}{V_{PCS} \cdot 2 \cdot F} + (J_{RyR2} + J_{IP3R}) \cdot \frac{V_{JSR}}{V_{PCS}} - J_{diff1})$$

- ***$[Ca^{2+}]_{SL}$***

$$\frac{d\left[Ca^{2+}\right]_{SL}}{dt} = -(I_{CaT} + I_{pCa} + I_{Cab} - 2 \cdot I_{NaCa,SL}) \cdot \frac{A_{Cap}}{V_{SL} \cdot 2 \cdot F} + J_{diff1} \cdot \frac{V_{SS}}{V_{SL}} - J_{SERCA,1} \cdot \frac{V_{NSR}}{V_{SL}} - J_{diff2}$$

$$TRPN_{SL} = \overline{TRPN_{SL}} \cdot \frac{[Ca^{2+}]_{SL}}{[Ca^{2+}]_{SL} + K_{m,TRPN}}$$

$$CMDN_{SL} = \overline{CMDN_{SL}} \cdot \frac{[Ca^{2+}]_{SL}}{[Ca^{2+}]_{SL} + K_{m,CMDN}}$$

$$[Ca^{2+}]_{SL,tot} = [Ca^{2+}]_{SL} + TRPN_{SL} + CMDN_{SL} + d[Ca^{2+}]_{SL}$$

$$b_{SL} = \overline{TRPN}_{SL} + \overline{CMDN}_{SL} - [Ca^{2+}]_{SL,tot} + K_{m,TRPN} + K_{m,CMDN}$$

$$c_{SL} = K_{m,TRPN} \cdot K_{m,CMDN} - [Ca^{2+}]_{SL,tot} \cdot \left(K_{m,TRPN} + K_{m,CMDN}\right) + \overline{TRPN_{SL}} \cdot K_{m,CMDN} + \overline{CMDN_{SL}} \cdot K_{m,TRPN}$$

$$d_{SL} = -K_{m,TRPN} \cdot c_{SL}\, K_{m,TRPN} \cdot [Ca^{2+}]_{SL,tot}$$

$$[Ca^{2+}]_{SL} = \frac{2}{3} \cdot \sqrt{{b_{SL}}^2 - 3 \cdot c_{SL}} \cdot \cos\left(\frac{1}{3}\cos^{-1}\left(\frac{9b_{SL}c_{SL} - 2{b_{SL}}^3 - 27d_{SL}}{2\left({b_{SL}}^2 - 3c_{SL}\right)^{1.5}}\right)\right) - \frac{b_{SL}}{3}$$

- ***[Ca^2+]i***

$$\frac{d\left[Ca^{2+}\right]_i}{dt} = \mathrm{J}_{diff2} \cdot \frac{\mathrm{V}_{SL}}{\mathrm{V}_{Myo}} - \mathrm{J}_{SERCA,2} \cdot \frac{\mathrm{V}_{NSR}}{\mathrm{V}_{Myo}} + \mathrm{J}_{RyR2} \cdot \frac{\mathrm{V}_{CSR}}{\mathrm{V}_{Myo}})$$

$$TRPN_{Myo} = \overline{TRPN_{Myo}} \cdot \frac{[Ca^{2+}]_i}{[Ca^{2+}]_i + K_{m,TRPN}}$$

$$CMDN_{Myo} = \overline{CMDN_{Myo}} \cdot \frac{[Ca^{2+}]_i}{[Ca^{2+}]_i + K_{m,CMDN}}$$

$$[Ca^{2+}]_{i,tot} = [Ca^{2+}]_i + TRPN_{Myo} + CMDN_{Myo} + d[Ca^{2+}]_i$$

$$b_{Myo} = \overline{TRPN}_{Myo} + \overline{CMDN}_{Myo} - [Ca^{2+}]_{i,tot} + K_{m,TRPN} + K_{m,CMDN}$$

$$c_{Myo} = K_{m,TRPN} \cdot K_{m,CMDN} - [Ca^{2+}]_{i,tot} \cdot \left(K_{m,TRPN} + K_{m,CMDN}\right) + \overline{TRPN_{Myo}} \cdot K_{m,CMDN} + \overline{CMDN_{Myo}} \cdot K_{m,TRPN}$$

$$d_{Myo} = -K_{m,TRPN} \cdot c_{Myo}\, K_{m,TRPN} \cdot [Ca^{2+}]_{i,tot}$$

$$[Ca^{2+}]_i = \frac{2}{3}\sqrt{{b_{Myo}}^2 - 3 \cdot c_{Myo}} \cos\left(\frac{1}{3}\cos^{-1}\left(\frac{9b_{Myo}c_{Myo} - 2{b_{Myo}}^3 - 27d_{Myo}}{2\left({b_{Myo}}^2 - 3c_{Myo}\right)^{1.5}}\right)\right) - \frac{b_{Myo}}{3}$$

- ***[Ca^2+]JSR***

$$\frac{d\left[Ca^{2+}\right]_{JSR}}{dt} = \mathrm{J}_{tr1} - \mathrm{J}_{RyR3} - \mathrm{J}_{IP3R}$$

$$CSQN_{JSR} = \overline{CSQN_{JSR}} \cdot \frac{[Ca^{2+}]_{JSR}}{[Ca^{2+}]_{JSR} + K_{m,CSQN}}$$

$$b_{JSR} = \overline{CSQN}_{JSR} + CSQN_{JSR} - [Ca^{2+}]_{JSR} + d[Ca^{2+}]_{JSR} + K_{m,CSQN}$$

$$c_{JSR} = K_{m,CSQN} \cdot (CSQN_{JSR} + [Ca^{2+}]_{JSR} + d[Ca^{2+}]_{JSR})$$

$$[Ca^{2+}]_{JSR} = \frac{\sqrt{{b_{JSR}}^2 - 4 \cdot c_{JSR}} - b_{JSR}}{2}$$

- ***[Ca^2+]CSR***

$$\frac{d\left[Ca^{2+}\right]_{JSR}}{dt} = \mathrm{J}_{tr2} - \mathrm{J}_{RyR2}$$

$$CSQN_{CSR} = \overline{CSQN_{CSR}} \cdot \frac{[Ca^{2+}]_{CSR}}{[Ca^{2+}]_{CSR} + K_{m,CSQN}}$$

$$b_{CSR} = \overline{CSQN}_{CSR} + CSQN_{CSR} - [Ca^{2+}]_{CSR} + d[Ca^{2+}]_{CSR} + K_{m,CSQN}$$

$$c_{CSR} = K_{m,CSQN} \cdot (CSQN_{CSR} + [Ca^{2+}]_{CSR} + d[Ca^{2+}]_{CSR})$$

$$[Ca^{2+}]_{CSR} = \frac{\sqrt{{b_{CSR}}^2 - 4 \cdot c_{CSR}} - b_{CSR}}{2}$$

- ***[Ca^2+]_NSR***

$$\frac{d\left[Ca^{2+}\right]_{NSR}}{dt} = \mathrm{J}_{SERCA,1} + \mathrm{J}_{SERCA,2} - \mathrm{J}_{tr1} \cdot \frac{\mathrm{V}_{JSR}}{\mathrm{V}_{NSR}} - \mathrm{J}_{tr2} \cdot \frac{\mathrm{V}_{CSR}}{\mathrm{V}_{NSR}}$$

- ***[Na^+]_SS***

$$\frac{d\left[Na^{+}\right]_{SS}}{dt} = -(\mathrm{I}_{CaNa} + 3 \cdot \mathrm{I}_{NaCa,SS}) \cdot \frac{\mathrm{A}_{Cap}}{\mathrm{V}_{SS} \cdot \mathrm{z}_{Na} \cdot \mathrm{F}} - \mathrm{J}_{diff1,Na}$$

- ***[Na^+]_SL***

$$\frac{d\left[Na^{+}\right]_{SL}}{dt} = -(3 \cdot \mathrm{I}_{NaK} + \mathrm{I}_{Na} + \mathrm{I}_{NaL} + \mathrm{I}_{NaCa,i} + \mathrm{I}_{fNa} + \mathrm{I}_{Nab}) \frac{\mathrm{A}_{Cap}}{\mathrm{V}_{SL}\mathrm{z}_{Na}\mathrm{F}} + \mathrm{J}_{diff1,Na} \frac{\mathrm{V}_{SS}}{\mathrm{V}_{SL}} - \mathrm{J}_{diff2,Na}$$

- ***[Na^+]_i***

$$\frac{d\left[Na^{+}\right]_{i}}{dt} = \mathrm{J}_{diff2,Na} \cdot \frac{\mathrm{V}_{SL}}{\mathrm{V}_{Myo}}$$

- ***[K^+]_SS***

$$\frac{d\left[K^{+}\right]_{SS}}{dt} = -\mathrm{I}_{CaK} \cdot \frac{\mathrm{A}_{Cap}}{\mathrm{V}_{SS} \cdot \mathrm{z}_{K} \cdot \mathrm{F}} - \mathrm{J}_{diff1,K}$$

- ***[K^+]_SL***

$$\frac{d\left[K^{+}\right]_{SL}}{dt} = -(\mathrm{I}_{to} + \mathrm{I}_{sus} + \mathrm{I}_{Kr} + \mathrm{I}_{Ks} + \mathrm{I}_{f,K} + \mathrm{I}_{K1} + \mathrm{I}_{stim} - 2 \cdot \mathrm{I}_{NaK}) \cdot \frac{\mathrm{A}_{Cap}}{\mathrm{V}_{SL} \cdot \mathrm{z}_{K} \cdot \mathrm{F}} + \mathrm{J}_{diff1,K} \frac{\mathrm{V}_{SS}}{\mathrm{V}_{SL}} - \mathrm{J}_{diff2,K}$$

- ***[K^+]_i***

$$\frac{d\left[K^{+}\right]_{i}}{dt} = -\mathrm{J}_{diff2,K} \frac{\mathrm{V}_{SL}}{\mathrm{V}_{Myo}}$$

6 - References

Bueno-Orovio, Alfonso, David Kay, and Kevin Burrage. 2014. "Fourier Spectral Methods for Fractional-in-Space Reaction-Diffusion Equations." *BIT Numerical Mathematics* 54 (4): 937–54. https://doi.org/10.1007/s10543-014-0484-2.

Deb, Kalyanmoy. 2001. *Multi-Objective Optimization Using Evolutionary Algorithms: An Introduction Multi-Objective Optimization Using Evolutionary Algorithms: An Introduction*. *Wiley-Interscience Series in Systems and Optimization*. New York, NY, USA.

Durrer, Dirk, R. Th Van Dam, G. E. Freud, M. J. Janse, F. L. Meijler, and R. C. Arzbaecher. 1970. "Total Excitation of the Isolated Human Heart," 899–912. https://doi.org/10.1161/01.CIR.41.6.899.

Dutta, Sara, Ana Mincholé, T. Alexander Quinn, and Blanca Rodriguez. 2017. "Electrophysiological Properties of Computational Human Ventricular Cell Action Potential Models under Acute Ischemic Conditions." *Progress in Biophysics and Molecular Biology* 129: 40–52. https://doi.org/10.1016/j.pbiomolbio.2017.02.007.

Gaborit, Nathalie, Sabrina Le Bouter, Viktoria Szuts, A. Varró, Denis Escande, Stanley Nattel, and Sophie Demolombe. 2007. "Regional and Tissue Specific Transcript Signatures of Ion Channel Genes in the Non-Diseased Human Heart." *The Journal of Physiology* 582 (Pt 2): 675–93. https://doi.org/10.1113/jphysiol.2006.126714.

Haissaguerre, Michel, Edward Vigmond, Bruno Stuyvers, Meleze Hocini, and Olivier Bernus. 2016. "Ventricular Arrhythmias and the His–Purkinje System." *Nature Reviews Cardiology* 13 (3): 1–12. https://doi.org/10.1038/nrcardio.2015.193.

Han, Wei, Liming Zhang, Gernot Schram, and Stanley Nattel. 2002. "Properties of Potassium Currents in Purkinje Cells of Failing Human Hearts." *American Journal of Physiology - Heart and Circulatory Physiology* 283 (6 52-6): H2495–2503. https://doi.org/10.1152/ajpheart.00389.2002.

Haufe, V., J. M. Cordeiro, T. Zimmer, Y. S. Wu, S. Schiccitano, K. Benndorf, and R. Dumaine. 2005. "Contribution of Neuronal Sodium Channels to the Cardiac Fast Sodium Current I Na Is Greater in Dog Heart Purkinje Fibers than in Ventricles." *Cardiovascular Research* 65 (1): 117–27. https://doi.org/10.1016/j.cardiores.2004.08.017.

Iyer, V., Danilo Roman-Campos, K. J. Sampson, Guoxin Kang, Glenn I Fishman, and Robert S Kass. 2015. "Purkinje Cells as Sources of Arrhythmias in Long QT Syndrome Type 3." *Scientific Reports* 5: 13287. https://doi.org/10.1038/srep13287.

Keener, J., and J. Sneyd. 2009. *Mathematical Physiology II: Systems Physiology*. Edited by Springer. 2nd ed.

Kupersmith, Joel, Ehud Krongrad, and Albert L. Waldo. 1973. "Conduction Intervals and Conduction Velocity in the Human Cardiac Conduction System." *Circulation* 47 (4): 776–85. https://doi.org/10.1161/01.CIR.47.4.776.

Li, Pan, and Yoram Rudy. 2011. "A Model of Canine Purkinje Cell Electrophysiology and Ca 2+ Cycling." *Circulation Research* 109 (1): 71–79.

https://doi.org/10.1161/CIRCRESAHA.111.246512.

Nagy, N., T. Szél, N. Jost, A. Tóth, J. Gy Papp, and A. Varró. 2015. “Novel Experimental Results in Human Cardiac Electrophysiology: Measurement of the Purkinje Fibre Action Potential from the Undiseased Human Heart.” *Canadian Journal of Physiology and Pharmacology* 810 (May): 1–8. https://doi.org/10.1139/cjpp-2014-0532.

O’Hara, Thomas, László Virág, András Varró, and Yoram Rudy. 2011. “Simulation of the Undiseased Human Cardiac Ventricular Action Potential: Model Formulation and Experimental Validation.” Edited by Andrew D. McCulloch. *PLoS Computational Biology* 7 (5): e1002061. https://doi.org/10.1371/journal.pcbi.1002061.

Pan, Li, and Yoram Rudy. 2011. “A Model of Canine Purkinje Cell Electrophysiology and Ca2+ Cycling: Rate Dependence, Triggered Activity, and Comparison to Ventricular Myocytes.” *Circulation Research* 109 (1): 71–79. https://doi.org/10.1161/CIRCRESAHA.111.246512.

Passini, Elisa, Ana Mincholé, Raffaele Coppini, Elisabetta Cerbai, Blanca Rodriguez, Stefano Severi, and Alfonso Bueno-Orovio. 2016. “Mechanisms of Pro-Arrhythmic Abnormalities in Ventricular Repolarisation and Anti-Arrhythmic Therapies in Human Hypertrophic Cardiomyopathy.” *Journal of Molecular and Cellular Cardiology* 96 (September): 72–81. https://doi.org/10.1016/j.yjmcc.2015.09.003.

Romero, L., E. Pueyo, M. Fink, and B. Rodriguez. 2009. “Impact of Ionic Current Variability on Human Ventricular Cellular Electrophysiology.” *AJP: Heart and Circulatory Physiology* 297 (4): H1436–45. https://doi.org/10.1152/ajpheart.00263.2009.

Rush, Stanley, and Hugh Larsen. 1978. “A Practical Algorithm for Solving Dynamic Membrane Equations.” *IEEE Transactions on Biomedical Engineering* BME-25 (4): 389–92. https://doi.org/10.1109/TBME.1978.326270.

Stewart, Philip, Oleg V. Aslanidi, Denis Noble, Penelope J. Noble, Mark R. Boyett, and Henggui Zhang. 2009. “Mathematical Models of the Electrical Action Potential of Purkinje Fibre Cells.” *Philosophical Transactions of the Royal Society A: Mathematical, Physical and Engineering Sciences* 367 (1896): 2225–55. https://doi.org/10.1098/rsta.2008.0283.

Stuyvers, Bruno D., Wen Dun, Scot Matkovich, Vincenzo Sorrentino, Penelope A. Boyden, and Henk E D J Ter Keurs. 2005. “Ca2+ Sparks and Waves in Canine Purkinje Cells: A Triple Layered System of Ca2+ Activation.” *Circulation Research* 97 (1): 35–43. https://doi.org/10.1161/01.RES.0000173375.26489.fe.

Tusscher, K. H. W. J. ten, and A. V. Panfilov. 2008. “Modelling of the Ventricular Conduction System.” *Progress in Biophysics and Molecular Biology* 96 (1–3): 152–70. https://doi.org/10.1016/j.pbiomolbio.2007.07.026.
